# Data Sources for Trait Databases: Comparing the Phenomic Content of Monographs and Evolutionary Matrices

**DOI:** 10.1371/journal.pone.0155680

**Published:** 2016-05-18

**Authors:** T. Alex Dececchi, Paula M. Mabee, David C. Blackburn

**Affiliations:** 1 Department of Biology, University of South Dakota, Vermillion, South Dakota, United States of America; 2 Florida Museum of Natural History, University of Florida, Gainesville, Florida, United States of America; Sars International Centre for Marine Molecular Biology, NORWAY

## Abstract

Databases of organismal traits that aggregate information from one or multiple sources can be leveraged for large-scale analyses in biology. Yet the differences among these data streams and how well they capture trait diversity have never been explored. We present the first analysis of the differences between phenotypes captured in free text of descriptive publications (‘monographs’) and those used in phylogenetic analyses (‘matrices’). We focus our analysis on osteological phenotypes of the limbs of four extinct vertebrate taxa critical to our understanding of the fin-to-limb transition. We find that there is low overlap between the anatomical entities used in these two sources of phenotype data, indicating that phenotypes represented in matrices are not simply a subset of those found in monographic descriptions. Perhaps as expected, compared to characters found in matrices, phenotypes in monographs tend to emphasize descriptive and positional morphology, be somewhat more complex, and relate to fewer additional taxa. While based on a small set of focal taxa, these qualitative and quantitative data suggest that either source of phenotypes alone will result in incomplete knowledge of variation for a given taxon. As a broader community develops to use and expand databases characterizing organismal trait diversity, it is important to recognize the limitations of the data sources and develop strategies to more fully characterize variation both within species and across the tree of life.

## Introduction

Over the past decade, the number of databases of organismal traits has grown substantially. These resources relate to many domains of biology, including studies of life histories [1, 2], genome sizes [3], developmental genetics and gene expression [4, 5], traits [6], and anatomical traits across the tree of life [7, 8]. As these phenotype resources expand and diversify, there is a rising need for ensuring that data from different domains are both computer readable and interoperable [9]. This interoperability creates discoveries, for example, by linking developmental genetics of model systems to phenotypes found in multiple species across the tree of life [8, 10, 11]. These discoveries are facilitated by both structured vocabularies (i.e., ontologies) and new data standards [12] that permit communication among diverse data sources. However, these databases also depend upon the creation of novel sets of curated and structured phenotype data for each domain of study.

To date, much of the ‘diversity’ data annotated in a computable format either is from or derives from matrices of anatomical characters used in phylogenetic analyses [8, 11, 13]. Phylogenetic matrices are a ready source of phenotypes because they are structured and information-dense. Moreover, they constitute a rarified data set: alternative states of phylogenetic characters are putative homologues and thus represent explicit hypotheses of genealogical relationships among taxa. Further, construction of phylogenetic matrices is focused on finding shared character states among taxa and not representing traits unique to a given taxon. The bulk of available phenotypes from the past two centuries, however, are not highly structured, standardized, or focused on phylogenetically informative traits. Instead, these descriptions are found in the free text of species descriptions [14] as well as anatomical, ethological, comparative, and even experimental studies. It is important to recognize the differences between these two sources of information on phenotypes even though they can refer to the same observable thing [15, 16]. Descriptions of morphological traits (morphemes sensu [16]), even when comparative, are not specifically articulating hypotheses of homology. In contrast, it is explicitly incorporated into the conceptualization of characters for phylogenetic analysis [17]. In many cases, it remains difficult to disentangle homologies from morphological descriptions, for instance when discussing the mesopodial elements. Because there is no quantitative evaluation of the types of phenotypes captured in these two important research outputs, it is not necessarily obvious to those creating trait databases whether there are meaningful differences between these data sources.

Because of the difference between describing morphology and characters, we expect that capturing phenotypes only from phylogenetic matrices will result in biases in the types of phenotypes populating newly developed species-level databases. For example, systematists exclude from phylogenetic analysis those traits thought to misleading or unimportant when inferring evolutionary relationships [18, 19]. This might include traits with high levels of homoplasy [20–22] and those thought to be strongly influenced by environmental factors [23]. Specific types of traits (e.g., coloration, texture, shape, behavior) should be underrepresented in matrices, including both anatomical entities and the qualities used to describe them. In addition, particular taxa might not be coded in most matrices, such as extinct species known only from partial fossils. These are sometimes not considered for analysis because of the many character states that would necessarily be coded as ‘missing’. For example, *Hynerpeton* Daeschler, Shubin, Thomason, & Amaral 1994 is an important Late Devonian taxon revealing important transitional forelimb morphology [24, 25], but is only recorded in a single matrix, due to the fragmentary material available. As the factors involved in convergent evolution are of high interest to a broad community of scientists [22, 26], the utility of community phenotypic databases, such as Phenoscape (http://kb.phenoscape.org; [27]), Traitbank (http://eol.org/info/516), or MorphoBank (http://www.morphobank.org), might be unintentionally limited by focusing on data sources that intentionally limit both homoplasy and traits unique to particular taxa (i.e., autapomorphies). While there are reasons to believe that phylogenetic matrices might not comprehensively capture phenotypic diversity within species and across the tree of life, we know of no attempt to quantify and compare differences between phenotypes found in free text (here, ‘monographs’) versus phylogenetic matrices (here, ‘matrices’). We also know of no previous study testing whether the characters created for phylogenetic analyses represent simply a subset of the morphological descriptions found, for example, in monographic treatments. Because of current large-scale efforts to create ‘data layers’ of traits across the tree of life (e.g., the Genealogy of Life initiative of the US National Science Foundation), exploration of this issue is timely.

Our goal was to evaluate potential biases in the data derived from free text descriptions and matrices by characterizing the ‘phenomic content’ of phenotypes in these two data sources. We characterize ‘phenomic content’ by calculating both the anatomical class space and the complexity of phenotypes in a given research product (i.e., a character matrix or morphological description). Specifically, we compared the anatomical entities, the qualities (i.e., size, shape, presence/absence), and the level of detail at which the anatomy was described. As a case study for these comparisons, we chose to focus on the evolutionary morphology of select taxa surrounding the fin-to-limb transition in early tetrapod vertebrates for two reasons. First, researchers have extensively described the anatomy of these taxa in both monographs and phylogenetic treatments. Some have focused on inferring evolutionary relationships by developing anatomically-based phylogenetic matrices. Others have taken a comparative and descriptive approach, often focusing on the anatomy that might have played a functional role in this evolutionary transition. Second, the existing Phenoscape Knowledgebase (http://kb.phenoscape.org/) is particularly enriched in the comparative skeletal anatomy for fins, limbs, and their support structures (girdles) of sarcopterygian vertebrates [13]. These well annotated taxa and phenotypes served as a rich source of computable phenotypes for this investigation. Our results provide baseline data for developing a strategy to create phenotype databases that maximize phenomic content and more comprehensively characterize known phenotypic diversity.

## Materials and Methods

### Phenotype Data Sources

We focused on phenotypes from the paired limbs/fins and girdles, which are well described anatomical regions in early tetrapods. We selected four extinct species that are well represented in both monographic and phylogenetic systematic treatments: *Acanthostega gunnari* Jarvik 1952, *Barameda decipiens* Woodward 1906, *Panderichthys rhombolepis* Gross 1941, and *Tiktaalik roseae* Daeschler, Shubin, & Jenkins 2006. These species represent different anatomical stages in the fin-to-limb transition from the late Devonian to the Early Carboniferous, are frequently used in phylogenetic analyses, and have recent and detailed descriptions of their limb/fin and girdle skeletons. For each taxon, 1–3 descriptive papers (‘monographs’; Table 1) were selected that focus on the paired limb/fin and/or girdle skeleton [28–33] or devote a significant discussion to these in the course of a longer monographic treatment [34]. Phylogenetic publications with curated matrices were selected from the Phenoscape Knowledgebase (KB) that included at least one of the four selected taxa, each of which contained at least 20 distinct characters (range: 20–155 characters) and 45 or more distinct character states (range: 45–387) for the limb/fin and girdle. These were created by different researchers or research groups with no overlap in authors and in no case was the taxon of interest stated to be an outgroup for the analysis (Table 1). This last criterion was used to minimize the impact of different character representation styles of individual investigators. In addition, we included sister publications dedicated to the anatomy of *Tiktaalik* [31, 35] to allow for direct comparison of phenotypic statements between monographic and matrix treatments by the same author set, written at the same time, and based on the same material. A benefit of focusing on skeletal morphology in monographic and phylogenetic treatments is that descriptions and characters are highly similar between extinct and extant vertebrates.

**Table 1:** List of monographic and matrix publications used in this analysis along with anatomical focus of the study and the number of fin or limb and girdle EQs (phenotypes) associated with each taxon.

Taxon	Monograph	Anatomical focus	# Monograph EQ	Matrix	# Matrix EQ
*Acanthostega gunnari*	Coates 1996	Whole body	341	Carroll 2007, Clack et al. 2012, Daeschler et al. 2006, Ruta 2011, Swartz 2012, Vallin and Laurin 2004	425
*Barameda decipiens*	Garvey et al. 2005	Pectoral fin	103	Ruta 2011, Swartz 2012	118
*Panderichthys rhombolepis*	Boisvert 2005, Boisvert et al. 2008, Boisvert 2009	Pelvic fin and girdle, Pectoral fin and girdle, Humerus	52, 51, 103 (total = 206)	Clack et al. 2012, Daeschler et al. 2006, Ruta 2011, Swartz 2012, Vallin and Laurin 2004	287
*Tiktaalik roseae*	Shubin et al. 2006, Shubin et al. 2014	Pectoral limb and girdle	117, 58 (total = 175)	Clack et al. 2012, Daeschler et al. 2006, Ruta 2011, Swartz 2012	226

### Phenotype Curation

Phenotypes from both monographs and matrices were composed using the Entity–Quality (EQ) formalism [36, 37] and Phenex software [38] as described previously [11]. Phenotypes were composed for all taxa referenced in each publication, though data from only the four taxa previously mentioned (Table 1) were analyzed. All annotations were done by TAD, for both monographs and matrix datasets, the former specifically for this analysis the later as part of larger Phenoscape project goals. Anatomical entities are represented by terms from the Uberon anatomy ontology for metazoan animals [39, 40] which is composed in part from independently developed multi-species vertebrate ontologies [41, 42]. Most entities in the Uberon anatomy ontology are ‘homology neutral’ in part because of the multiple axes of classification for entities including structure, function, and development. This maintains “biologically informative” linkages in entities across different organisms without imposing restrictions related to phylogenetic relationships. For example, because the entity “eye” (UBERON:0000970) is defined as an “organ that detects light”, this term is used across metazoans for light-collecting sensory organs regardless of their homology. The recent incorporation of expertly vetted homology statements, e.g., from Bgee (database for Gene Expression Evolution; [43]) into Uberon in the Vertebrate Homologous Organ Group Ontology (vHOG; [44]) and using the property “homologous _to” makes it possible for users to make explicit homology statements when annotating characters or morphological descriptions [40]. While we did add some anatomical entities to Uberon during curation, most terms were already available in this ontology. Some anatomy ontology terms were ‘post-composed’, meaning that terms from one or more ontologies were combined to create a new term [11, 37]. Frequently this involved terms for the processes, margins, and regions of specific structures. For example, to represent the anatomical structure “proximal region of the pectoral fin ray” we use the term ‘proximal region’ from the spatial ontology (BSPO:0000077) and ‘pectoral fin lepidotrichium’ from the anatomy ontology (UBERON:4000175) to create the post-composed term ‘proximal region part_of some (pectoral fin lepidotrichium)’. Generally, terms are post-composed when they are not regularly used in the literature [41]. Phenotypic qualities (e.g., presence/absence, size, shape, composition) are taken from the Phenotype and Trait Ontology (PATO; [45]). Terms for taxonomic names of vertebrate taxa are from the Vertebrate Taxonomy Ontology (VTO; [46]).

Phenotypes from monographs were constructed using a slightly modified annotation procedure relative to the characters from phylogenetic matrices [41]. Specifically, we annotated ‘phenotypic statements’, i.e., complete anatomical descriptions made by an author regarding a phenotype. These may include multiple anatomical elements and multiple lines of text in the original description. A phenotypic statement may reference more than one taxon and describe morphological variation among them. We refer to those phenotypic statements that are direct comparisons asserted between multiple taxa in a monograph as ‘comparative statements’. Phenotypic statements range from simple declarative statements such as “This suggests that the fin fanned out and would have been paddle-shaped” (Garvey et al. 2005 [30], p. 14) to longer descriptions such as “Although the mesial surface of the glenoid is thickened, there is no evidence of a well ossified ventral footing which could constitute a substantial infraglenoid buttress. This absence of an infraglenoid buttress is unique among limb-bearing scapulocoracoids, and makes a striking contrast with those of *Ichthyostega* Säve-Söderbergh 1932 (Jarvik, 1980, 1996 [47, 48]), *Tulerpeton* Lebedeu 1984 (Lebedev & Coates 1995 [49]), *Hynerpeton* (Daeschler et al. 1994 [24]), and post-Devonian tetrapods (Coates, 1996 [34] pp. 379–380).” Annotating phenotypic statements using the EQ format allowed for direct comparisons to matrix-based phenotypes.

We limited our study to a subset of vertebrates along the fin to limb transition, focusing on making comparisons between four key taxa. This was our focus because of the richness of phylogenetic and monographic data available for each taxon, the relevant expertise of the curators involved in Phenoscape, and the considerable curation effort involved. In addition, because these are extinct vertebrates known nearly entirely from their skeletons, one might expect more comparability in the phenotypes and anatomical terms used in matrices and monographs than for extant taxa for which other anatomical systems might be studied. Dahdul et al. [50] recently estimated the rate of ontology-enabled curation of phylogenetic matrices by trained experts. Using their highest estimate (13.5 characters/hour) and assuming monograph annotation takes roughly the same amount of time, our curation of these monographic datasets took approximately 27 hours. Using the same measure, the matrix datasets involved approximately 25 hours. However, because our curation effort also required the creation of some new ontology terms, our rate of curation was significantly less than the maximum estimate from Dahdul et al. [50] and likely places the amount of curation effort closer to 100 hours total.

### Analysis of Phenomic Content

We used three different measures to quantify the phenomic content from evolutionary matrices versus monographs. These included the level of anatomical detail captured in phenotype descriptions, the particular classes of qualities used to describe phenotypes, and the relative amounts of anatomical class space covered by the anatomical entities used in each data source.

We compared the level of anatomical detail (i.e., granularity of phenotypes) from matrix-based characters to those from monographic statements by calculating EQ complexity, which is defined simply as the number of classes and properties used in an EQ statement [51]. For example, the annotation from Swartz [52] of E: opercle, Q: absent has only two classes in it, one for the entity and one for the quality. Therefore, the EQ complexity is 2. In contrast, the annotation from Daeschler et al. [35] of E: anatomical projection part_of some (dorsal surface^part_of some (scapulocoracoid)), Q: attached_to RE (Related Entity): cleithrum, has seven classes and an EQ complexity of 7. EQ complexity provides an estimate of the level of detail of the anatomical description because higher levels of post-composition (i.e., more classes) are required for more fine-grained differentiation of features. To be clear, we are focused on the complexity of the description used by authors not complexity of the morphology itself.

To characterize and quantify the qualities (Q) of phenotypes derived from each publication, we classified these according to four formal classes, i.e., four high-level quality ontology terms. These classes are generally consistent with the kinds of qualitative and quantitative transformational morphological characters outlined by Sereno [53]. They are: (1) Presence/Absence, which we will refer to as ‘Neomorphic’, represented by two subtypes of ‘amount’ (PATO:0000070)–‘present’ (PATO:00000467) and ‘absent’ (PATO:0000462); (2) Morphology, ‘morphology’ (PATO:0000051), and its descendent terms ‘shape’ (PATO:0000052), ‘size’ (PATO:0000117), ‘texture’ (PATO:0000150), and ‘structure’ (PATO:0000141); (3) Position, ‘position’ (PATO:0000140) and its subtypes (e.g., ‘orientation’); and (4) Number, ‘amount’ (PATO:0000070). These terms and their formal definitions and relationships can be viewed in a web-based ontology viewer (e.g., http://www.ontobee.org/; http://bioportal.bioontology.org). While determining whether a phenotype is truly ‘neomorphic’ depends a phylogenetic perspective, many authors writing morphological descriptions specifically note whether a phenotype is present or absent in the taxon of interest. We use the term ‘Neomorphic’ as a shorthand to represent Presence/Absence phenotypes from either data source.

To compare entities (E) between monographs and matrices, we extracted all named anatomical entities that had ‘part_of’ relationships to ‘paired limb/fin’ (UBERON:0004708) or ‘girdle skeleton’ (UBERON:0010719) from the EQs in each publication, including those from post-compositions and related entity (RE) statements. We did not include generalized anatomical terms (i.e., unnamed features) such as ‘anatomical projection’ or ‘bone fossa’. Entities were concatenated from matrix and monograph publications. The intersection and differences of term lists from these sets were calculated manually. The number of fin, limb, and girdle classes was calculated from the query to Uberon (as of 25 January 2016): “(part_of some Skeletal System) and (part_of some Appendage Girdle Complex).” This query used the ELK OWL reasoner [54] that takes transitivity and other logical inferences into account. There are 1,216 entities and related parts that are children of either ‘paired limb/fin’ or ‘girdle skeleton’ in the Uberon anatomy ontology. These 1,216 entities comprise the maximum possible number relevant to these two anatomical regions. Comparing the entities used in curation of matrices and monographs provides a coarse measure of their respective phenomic content.

A report listing taxa and associated EQs from the Phenoscape Knowledgebase (S1 Appendix) was used to (1) compare the overlap of entities (E) and phenotypes (EQs) for monographs and matrices for each of the four taxa, and (2) calculate the anatomical entities (E), qualities (Q), and phenotypes (EQ) that were unique to monographs and matrices. To assess the level of congruence between assertions in matrix vs. monograph, the OntoTrace tool [13] was used to compile a synthetic supermatrix of presence/absence characters for the limb/fin and girdle entities in the matrix and monograph files.

## Results

**Fig 1:**
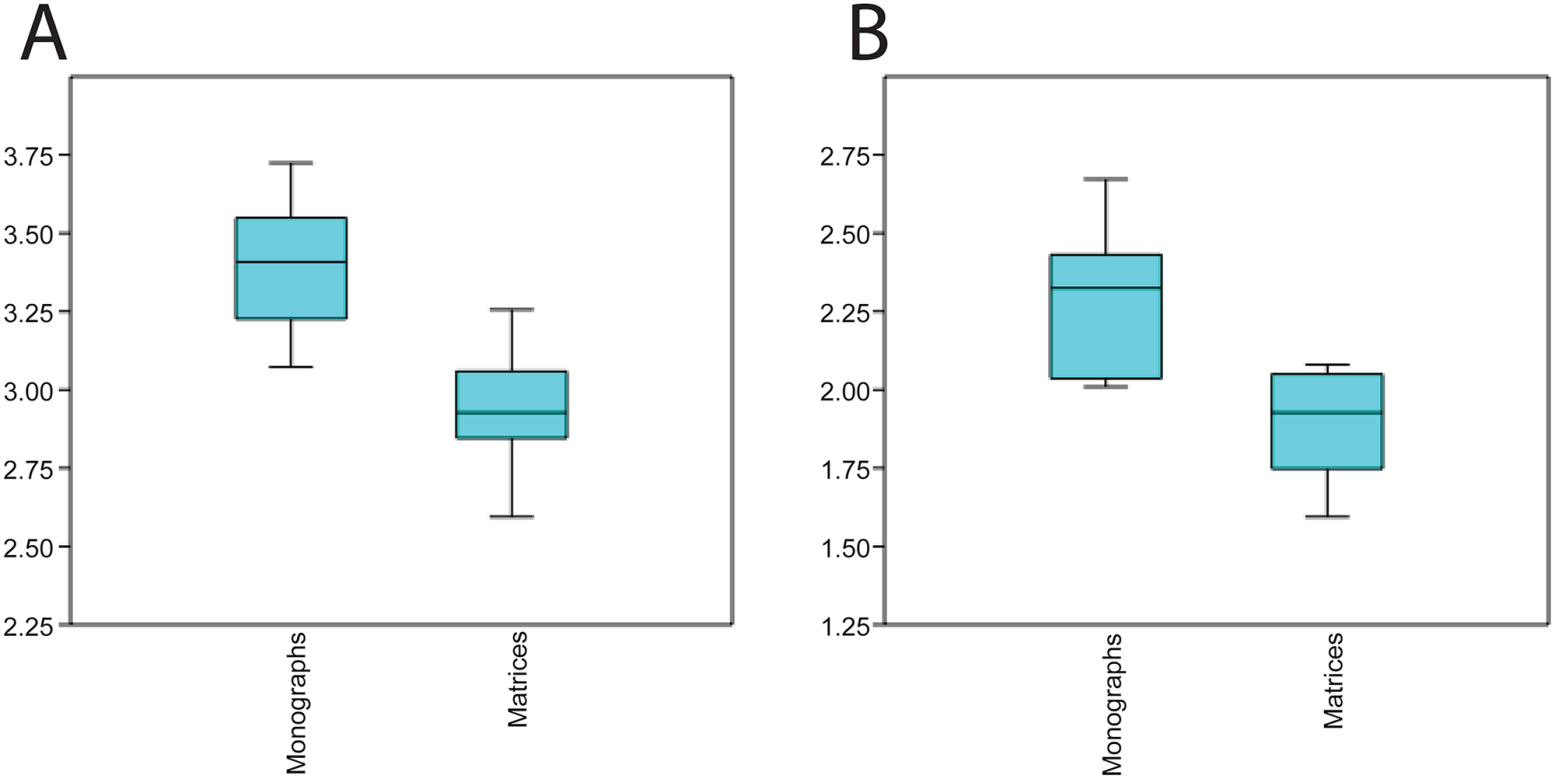
Boxplots showing comparison of mean EQ (A) and E (B) size per annotation between monographs and matrices. See Table 2 for details. Comparison of quality size was not included as there was no significant difference between mean quality complexity between the two sources of data (t-test p = 0.18). Breakdown of types per publication shown in S2 Appendix.

The number of descriptions and character statements from each monograph and matrix publication, respectively, were similar (monographs: mean 52.1, range 19–158; matrices: mean 56.2, range 19–155; Fig 1). However, the resulting number of EQs corresponding to these statements was higher in matrices (monographs: mean 119.3, range 51–341; matrices: mean 215.8, range 69–500). Except for one study [32], matrices always included more taxa than monographs (Tables 2 and 3). For each of the four taxa, the combined matrix dataset contained a larger number of entities than found in the monographs (Table 1). Matrices also contained a larger proportion of unique entities relative to the total number of entities (Table 4), yet there was no significant difference between the percentage of unique entities of monographs and matrices overall (Table 4). In comparing phenotypes for a given taxon, the overlap of entities between monographs and matrices was low, ranging from 23% to 49% (Table 4).

**Table 2:** Breakdown per monographic publication of the number of phenotype statements, percentage of comparative statements with the absolute number of statements given in parentheses, number of limb/fin and girdle EQ annotations, number of taxa referenced in the limb/fin and girdle section of the monograph, and the average EQ complexity (see Methods).

Publication	Phenotype statements	No. Comp. statements	EQs	Taxa	EQ complexity (mean, med., max.)	E (mean, max.)	Q (mean, max.)
Boisvert 2005	19	2	52	3	3.1, 3, 7	2.0, 6	1.0, 3
Boisvert et al. 2008	22	5	51	4	3.6, 3, 9	2.4,7	1.1, 3
Boisvert 2009	42	20	103	11	3.3, 3, 7	2.0, 6	1.3, 5
Coates 1996	158	15	341	18	3.4, 3, 11	2.4, 10	1.0, 5
Garvey et al. 2005	54	10	113	10	3.7, 3, 15	2.7, 14	1.1, 3
Shubin et al. 2006	46	8	117	9	3.5, 3, 11	2.3,10	1.1, 3
Shubin et al. 2014	24	7	58	6	3.2, 3, 7	2.1, 6	1.1, 3
**Mean**	**52.1**	**9.6**	**119.3**	**8.7**	**3.4**	**2.3**	**1.1**

**Table 3:** Breakdown per matrix publication of the number of characters and states, limb/fin and girdle EQ annotations, taxa referenced in the limb/fin and girdle section of the monograph, and the average EQ complexity (see Methods). Char. = Character; Char. States = Character States.

Publication	Char.	Char. States	EQs	Taxa	EQ complexity (mean, med., max.)	E (mean, max.)	Q (mean, max.)
Carroll 2007	49	199	422	22	3.1, 2, 11	2.1, 10	1.0, 3
Clack et al. 2012	19	43	69	22	2.6, 2, 6	1.6, 5	1.0, 1
Daeschler et al. 2006	32	67	85	9	2.9, 2, 7	1.9, 6	1.0, 1
Ruta 2011	155	393	500	44	3.3, 2, 10	2.1, 9	1.2, 7
Swartz 2012	46	96	123	47	2.9, 2, 6	1.8, 5	1.1, 5
Vallin and Laurin 2004	36	89	96	49	2.9, 2, 6	1.9, 5	1.0, 1
**Mean**	**56.2**	**147.8**	**215.8**	**32.2**	**2.9**	**1.9**	**1.1**

**Table 4:** Total number of girdle and limb anatomical entities, unique and shared, described in monographs vs. matrices for each of the study taxa.

Taxon	Total Entities monograph	Total Entities matrix	Unique Entities in monograph	Unique Entities in matrix	Number shared E and total E
*Acanthostega*	117	145	31/117 (26%)	59/145 (41%)	86/176 (49%)
*Barameda*	30	53	9/30 (30%)	32/53 (60%)	21/62 (34%)
*Panderichthys*	67	86	38/67 (57%)	57/86 (67%)	29/124 (23%)
*Tiktaalik*	42	68	21/42 (50%)	47/68 (68%)	21/89 (24%)
**TOTAL**	**178**	**154**	**71/178 (40%)**	**49/154 (32%)**	**107/226 (47%)**

For *Tiktaalik*, we compared limb/fin phenotypes between monographic and matrix treatments by the same author set, based on the same material, and published together [31, 35]. We found that 50% of the annotations in the matrix publication were not present in the companion monograph, and 74% of the annotations in the monograph were not present in the companion matrix publication (Table 5).

**Table 5:** Number of anatomical entities, unique and shared, described in monographs vs. matrices for *Tiktaalik*.

	Total Entities Monograph	Total Entities Matrix	Unique Entities in monograph	Unique Entities in matrix	Intersection of Entities
Daeschler et al. 2006	-	22	-	11 (50%)	11
Shubin et al. 2006	42	-	31 (74%)	-	11

Comparing the entities used in the works studied here, the anatomical class space of monographic treatments (178/1216; 14.6%) is approximately the same as that of matrices (154/1216; 12.7%). Together, the 228 total anatomical classes used to describe phenotypes in these two data sources cover approximately 18.8% of the potential class space to describe fin and limb skeletal anatomy represented in the Uberon anatomy ontology (see S4 Appendix).

A comparison of phenotype qualities showed that monographs include significantly more ‘morphology’ (PATO:0000051) and ‘position’ (PATO:0000140) phenotypes, whereas matrices include significantly more Neomorphic characters (Table 6; S2 Appendix). In fact, the ranges of Neomorphic characters did not overlap between monographs and matrices.

**Table 6:** Average percent of character quality types (range in parentheses) for matrix publications (combined) and monographs (combined). t-test *p<0.05; **p<0.001. Breakdown of types per publication shown in S2 Appendix.

Character quality type	Matrix	Monograph
Morphology	36.8 (17.8–48.4)	54.3* (30.2–70.4)
Neomorphic	37.2 (24.1–46.5)	12.4** (0.0–16.7)
Position	16.0 (9.9–26.4)	30.5* (13.0–51.9)
Number	10 (0.0–28.0)	2.8 (0.0–7.4)

The average EQ complexity of phenotypes found in monographs was significantly greater than that in matrices (3.4 and 2.9, respectively; t-test, *p*-value 0.003; Tables 2 and 3). In general, the range of EQ complexity was greater in monographs (range: 2–15) than matrices (range: 2–11) and the average number of entities used in EQs of each monograph was mostly greater than those used in matrices (Tables 2 and 3). The minimum EQ complexity (2) was identical among all monographs and matrices examined. While EQ complexity scores of 2, 3, and 4 represented the bins with the largest proportions of characters in each study, there were generally fewer EQ complexity scores of 3 in matrices (monograph mean: 18%, range 11–27%; matrix mean: 9%, range 3–13%). When examined in detail, nearly all EQs with complexity scores of 3 detailed either the relative size or positional relationship between two entities, a relatively common feature of descriptions in monographic treatments.

No presence/absence conflicts were detected for entities described in monographic treatments. However, conflicting statements were identified between entities in matrices and between matrices and monographs (number of conflicts: *Acanthostega*, 13; *Barameda*, 0; *Panderichthyes*, 7; *Tiktaalik*, 5). For instance, the scapular blade was asserted to be absent in matrices [52, 55] but present according to the monographic treatment of Coates [34]: “In *Acanthostega* sections through the base of the cleithrum/ scapular blade reveal a striking pattern of highly vascularised dermal bone-like histology flanking more broadly trabecular endochondral bone.” In another example, the postaxial process of the fibula was inferred present in *Panderichthys* based on the statement “Postaxial process on fibula size: small” [52], but asserted absent in both a matrix [56] and a monograph [29].

## Discussion

This first baseline study of phenomic content of monographs and matrices revealed subtle but important differences between these data sources. Matrices are not simply a ‘phylogenetically informative subset’ of the traits discussed in free text descriptions of monographs. For instance, there is less than a 50% overlap between the anatomical classes used to describe traits for each taxon between monographs and matrices (Table 4). Taken together, these two data sources provide a richer characterization of phenotypic diversity than either would individually. In general, text descriptions in monographs reference fewer taxa, focus more on traits represented by ‘morphology’ and ‘position’ terms from PATO, and feature somewhat more complex phenotype descriptions. While matrices are inherently comparative and thus include comparisons among more taxa, the conceptualization of these is simpler. This suggests a general and perhaps unsurprising trade-off between making detailed descriptive phenotypes and surveying for homologous phenotypes across many species. These results are consistent with what might be expected based on the conceptual differences between describing morphology (monographs) and characters (matrices) [15]. Yet even in combination, these two data sources represent only a subset of all possible phenotypes, as demonstrated by the fact that all of the anatomical classes included in this analysis cover less than 20% of those possible for the limb/fin in Uberon.

The higher percentage of unique anatomical entities represented in phenotypes from the matrix dataset versus monographs (Table 4) results from several factors. First, for each taxon, multiple matrices were combined (Table 1) to calculate the unique entity list resulting in a large number of combined characters. Second, most of the matrix publications post-date the associated monographs (Table 1) and thus were able to incorporate information from these monographs. In addition, phylogenetic studies published after the monographs have the opportunity to examine new specimens and re-interpret specimens in the process of coding and writing character descriptions. Yet when directly comparing entities used in monograph and matrix sister publications [31, 35], this trend is reversed (Table 5), with the monographic description using overall more anatomical classes (42 vs. 22) and more unique ones (74% vs. 50%) than the phylogenetic characters included in the matrix. Another possibility is that matrices may more often note phenotypes that are missing or absent. It may be acceptable for a free text description to omit mention of entities that are missing because a fossil is incomplete, but the format of a matrix necessitates that the entity is ‘mentioned’ by being coded as either missing or absent. If an anatomical entity is noted explicitly as ‘absent’, we consider that to be phenomic content, but not when it is explicitly noted as ‘missing’ (often coded as ‘?’) data. However, this can be difficult to disentangle if an author conflated absent and missing when coding their phylogenetic matrix. Removing such entities from our calculations for matrices is unlikely to fundamentally alter our results. For example, of the 32 unique entities found in matrices for *Barameda* (Table 4), only nine of these are for traits mentioned as absent. The conflation of ‘missing’ and ‘absent’ as well as ‘small’ or ‘reduced’ and ‘absent’ (see example in Results) may mislead analyses utilizing these data sources and are important to address when creating trait databases.

The qualities of phenotypes differ between monographs and matrices. Matrices include significantly more Neomorphic phenotypes, and monographs include significantly more phenotypes with qualities related to Morphology (‘morphology’ [PATO:0000051] and descendent terms) and Position (‘position’ [PATO:0000140] and descendent terms) (see Table 6). The higher percentage of Neomorphic phenotypes in matrices results in part from a preference for characters with low homoplasy for phylogenetic analyses [18] as well as an ease of coding diverse taxa for a given character (presence or absence being simpler than, e.g., round or ovoid).

Phenotype statements from monographs have a marginal tendency to higher EQ complexity. This is not surprising as a somewhat higher EQ complexity reflects the purpose of these treatments to comprehensively describe traits preserved instead of only phylogenetically informative ones. However, at a coarser level, the EQ complexity of phenotype statements is generally similar between monographs and matrices. For example, the vast majority of phenotype and character statements of monographs and matrices, respectively, have EQ complexity scores of 2, 3, and 4, which represent 76–87% of the EQs in monographs and 81–96% of those in matrices (S3 Appendix). While the maximum EQ complexity for an individual work may range from 6 to 15 (Tables 2 and 3), EQ complexity scores of 10 or higher are uncommon (only 7 of 842 EQs for monographs, and 4 of 1295 EQs for matrices).

The tendency towards a greater number of EQs of minimal complexity (2) in matrices derives in part from a focus on Neomorphic characters (Table 3) that are represented as the presence or absence of a specific entity. In contrast, the somewhat higher level of phenotypic complexity in monographs may be related to a greater emphasis on characters related to function. Beside the issues of homoplasy, there is also the issue of integration and avoidance of correlated characters that may help partially explain this. For example, the elbow joint is described as a single character ‘radial facets’ with two states (‘faces distally’, ‘has some ventrally directed component’) in Daeschler et al. [35]. In contrast, in the free text description of Shubin et al. [31], the function of the radial facet is described in two paragraphs relating to seven phenotype statements. These include relative size differences between the radial and ulnar facets, position of one relative to the other, and the fact that the radial facet is a convex, bent ellipsoid. These statements are then synthesized into a series of statements related to the function of the elbow joint.

A broadly used and well-provisioned anatomy ontology can be used as a proxy for the full set of knowledge of fin and limb anatomy. Against this, the proportion of anatomy class space, i.e., the proportion of terms used in a dataset (e.g., monograph, matrix) versus all relevant anatomical terms, can be compared. The anatomy ontology used here (Uberon; [40]) contains a total of 1,216 fin, limb, and girdle skeletal classes relevant to vertebrates. This knowledge was assembled through the long-term annotation efforts of a broad community encompassing biomedical and biodiversity domains. For example, the class ‘digit’ was required to annotate mouse phenotypes resulting from genetic manipulation and ‘lepidotrichium’ was added to annotate zebrafish phenotypes. A focused effort by the Phenoscape project (www.phenoscape.org) to annotate fin and limb characters from over 55 matrices [13] resulted in the addition of terms such as ‘scapular blade’ and ‘manual digit 8’. Thus one might consider the knowledge of the fins, limbs, and their supporting girdles to be more fully represented than any other anatomical region. We found, however, that the terms used in this literature to describe limb/fin and girdle phenotypes cover only 19% of the possible classes for fin and limb skeletal anatomy.

While limited to a small group of focal taxa and a limited set of anatomical features, our analyses suggest that extracting phenotypes from multiple data sources is required to comprehensively represent organismal anatomy when creating trait databases. Important aspects of phenomic content differ between these two data sources including differences in the way that phenotypes are represented and the general types of qualities and specific sets of anatomical entities used. While there is a tendency to greater complexity of phenotype statements in the free text descriptions in monographs, these statements are nearly always among fewer taxa. Depending on the use of phenotype data, authors may have preferences for richer descriptive information for entities or simply phylogenetically informative phenotypes sampled across more taxa. Databases representing diversity across the tree of life must make a concerted effort to mine diverse data sources, such as evolutionary matrices and free text descriptions, to generate more comprehensive assessments of phenotypic knowledge. By sampling phenotypes from both matrices and free text, our phenotype databases will better serve the communities working across diverse domains of biology.

## Supporting Information

S1 Appendix Reports of entities and qualities used in curation of EQ statements for each taxon. (DOCX)

S2 Appendix Percent character types in individual matrices and monographs. (DOCX)

S3 Appendix Breakdown of EQ complexity scores across EQ statements from matices and monographs. (DOCX)

S4 Appendix Entities describing all limb/fin skeleton class space compared to those used in curation of EQ statements of matrices and monographs. (XLSX)
