# Supplementary material for: Data Sources for Trait Databases: Comparing the Phenomic Content of Monographs and Evolutionary Matrices
Source: PLoS One. 2016 May 18;11(5):e0155680. doi: 10.1371/journal.pone.0155680 (PMC4871461; doi:10.1371/journal.pone.0155680)
Supplement: S1 Appendix — (DOCX) [file pone.0155680.s001.docx]

**Appendix S1.**

The following reports were generated for each taxa based on the curated matrices and ‘monographs’ from the Phenoscape Knowledgebase. These reports list the entities and qualities used in each EQ statement from these works. These data were used to (1) compare the overlap of entities (E) and phenotypes (EQs) for monographs and matrices for each of the four taxa, and (2) calculate the anatomical entities (E), qualities (Q), and phenotypes (EQ) that were unique to monographs and matrices.

**EQs from Matrices for *Acanthostega***

|  | Entities | Qualities | Related Entities |
| --- | --- | --- | --- |
| Carroll 2007 | [anatomical conduit and (part_of some humerus)] | present |  |
|  | [anatomical conduit and (part_of some puboischiadic plate)] | count |  |
|  | [anatomical projection and (part_of some (diaphysis and (part_of some ulna)))] | increased size |  |
|  | [anatomical projection and (part_of some (dorsal region and (part_of some iliac blade)))] | bifurcated |  |
|  | [anatomical projection and (part_of some (ventral region and (part_of some radius bone)))] | present |  |
|  | [anatomical projection and (part_of some ectepicondyle of humerus)] | present |  |
|  | [anatomical projection and (part_of some entepicondyle of humerus)] | increased size |  |
|  | [anatomical projection and (part_of some entepicondyle of humerus)] | posterior orientation |  |
|  | [anatomical surface and (part_of some acetabular part of hip bone)] | structure |  |
|  | [articular surface and (part_of some (glenohumeral joint and (part_of some humerus)))] | coiled |  |
|  | [articular surface and (part_of some humeroulnar joint)] | separated from | [articular surface and (part_of some humeroradial joint)] |
|  | [articular surface and (part_of some humeroulnar joint)] | ventrolaterally orientation |  |
|  | [articulation and (part_of some (femur and (part_of some acetabular part of hip bone)))] | associated with | [proximal region and (part_of some femur)] |
|  | [articulation and (part_of some (femur and (part_of some acetabular part of hip bone)))] | broad |  |
|  | [articulation and (part_of some (proximal region and (part_of some femur)))] | acute angle to | [articulation and (part_of some (distal region and (part_of some femur)))] |
|  | [diaphysis proper and (part_of some tibia)] | decreased size |  |
|  | [diaphysis proper and (part_of some tibia)] | flat |  |
|  | [endochondral element and (part_of some (ventro-medial region and (part_of some pectoral girdle skeleton)))] | absent |  |
|  | [fibula and (bearer of some torsioned)] | angle |  |
|  | [individual digit of digitopodial skeleton and (part_of some manus)] | count |  |
|  | [individual digit of digitopodial skeleton and (part_of some pes)] | count |  |
|  | [lateral margin and (part_of some tibia)] | overlapped by | fibula |
|  | [lateral margin and (part_of some tibia)] | straight |  |
|  | [lateral region and (part_of some (diaphysis and (part_of some fibula)))] | [shape and (not (concave))] |  |
|  | [medial region and (part_of some (diaphysis and (part_of some fibula)))] | concave |  |
|  | [phalanx and (part_of some manual digit 1 digitopodial skeleton)] | count |  |
|  | [phalanx and (part_of some manual digit 2 digitopodial skeleton)] | count |  |
|  | [phalanx and (part_of some manual digit 3 digitopodial skeleton)] | count |  |
|  | [phalanx and (part_of some manual digit 4 digitopodial skeleton)] | count |  |
|  | [phalanx and (part_of some manual digit 5 digitopodial skeleton)] | count |  |
|  | [phalanx and (part_of some manual digit 6 digitopodial skeleton)] | count |  |
|  | [phalanx and (part_of some manual digit 7 digitopodial skeleton)] | count |  |
|  | [phalanx and (part_of some manual digit 8 digitopodial skeleton)] | count |  |
|  | [phalanx and (part_of some pedal digit 1 digitopodial skeleton)] | count |  |
|  | [phalanx and (part_of some pedal digit 2 digitopodial skeleton)] | count |  |
|  | [phalanx and (part_of some pedal digit 3 digitopodial skeleton)] | count |  |
|  | [phalanx and (part_of some pedal digit 4 digitopodial skeleton)] | count |  |
|  | [phalanx and (part_of some pedal digit 5 digitopodial skeleton)] | count |  |
|  | [phalanx and (part_of some pedal digit 6 digitopodial skeleton)] | count |  |
|  | [phalanx and (part_of some pedal digit 7 digitopodial skeleton)] | count |  |
|  | [phalanx and (part_of some pedal digit 8 digitopodial skeleton)] | count |  |
|  | [tibia and (bearer of some torsioned)] | absent |  |
|  | acetabular part of hip bone | anterior to | [ventral region and (part_of some iliac blade)] |
|  | adductor blade | increased length |  |
|  | adductor crest | extends to | [distal region and (part_of some diaphysis of femur)] |
|  | adductor crest | increased length |  |
|  | anocleithrum | present |  |
|  | centrale | absent |  |
|  | clavicle | present |  |
|  | cleithrum | fused with | scapula |
|  | cnemial crest | present |  |
|  | distal tarsal bone | count |  |
|  | ectepicondylar foramen | present |  |
|  | entepicondylar foramen | present |  |
|  | epiphysis | increased size |  |
|  | fibulare | circular |  |
|  | fibulare | rectangular |  |
|  | fourth trochanter | elongated |  |
|  | glenoid fossa | coiled |  |
|  | glenoid fossa | strap-shaped |  |
|  | hindlimb intermedium | circular |  |
|  | hindlimb intermedium | rectangular |  |
|  | humerus | L-shaped |  |
|  | humerus | flattened |  |
|  | humerus diaphysis | absent |  |
|  | ilium | fused with | pubis |
|  | ilium | fused with | ischium |
|  | interclavicle | present |  |
|  | intermedium (fore) | present |  |
|  | internal trochanter | proximal to | head of femur |
|  | intertrochanteric fossa | increased size |  |
|  | intertrochanteric fossa | anterior orientation |  |
|  | ischium | fused with | pubis |
|  | latissimus dorsi process | present |  |
|  | medial tibial tarsal bone | circular |  |
|  | medial tibial tarsal bone | rectangular |  |
|  | olecranon | absent |  |
|  | postbranchial lamina | present |  |
|  | proximal epiphysis of humerus | acute angle to | distal epiphysis of humerus |
|  | puboischiadic plate | increased size |  |
|  | scapula | fused with | coracoid bone |
|  | scapulocoracoid | perforate |  |
|  | supinator process | fused with | deltopectoral crest |
|  | tibia | increased length | fibula |
|  | tibia | unfused from | fibula |
|  | ulna | unfused from | radius bone |
|  | ulna | decreased length | radius bone |
| Clack et al. 2012 | [anatomical conduit and (part_of some iliac neck)] | absent |  |
|  | [anatomical conduit and (part_of some ventral humeral ridge)] | absent |  |
|  | [anatomical conduit and (part_of some ventral humeral ridge)] | decreased size |  |
|  | [articulation and (part_of some (anterior side and (part_of some anocleithrum)))] | absent |  |
|  | [proximal region and (part_of some ventral humeral ridge)] | absent |  |
|  | anocleithrum | teardrop-shaped |  |
|  | cleithrum | unornamented |  |
|  | coracoid foramen | absent |  |
|  | deltopectoral crest | present |  |
|  | entepicondylar foramen | present |  |
|  | entepicondyle of humerus | square |  |
|  | entepicondyle of humerus | parallelogram |  |
|  | individual digit of digitopodial skeleton | present |  |
|  | interclavicle | increased size |  |
|  | interclavicle | exposed |  |
|  | interclavicle | diamond shaped |  |
|  | intertemporal bone | absent |  |
|  | latissimus dorsi process | present |  |
|  | pectoral fin lepidotrichium | absent |  |
|  | pelvic fin lepidotrichium | absent |  |
|  | postbranchial lamina | present |  |
|  | posterior process of ilium | posterodorsal orientation |  |
|  | posttemporal | absent |  |
|  | prepectoral space | absent |  |
|  | radius bone | increased length | ulna |
|  | scapular blade | absent |  |
|  | scapulocoracoid | increased size |  |
|  | scapulocoracoid | platelike |  |
|  | subscapular fossa | decreased depth |  |
|  | subscapular fossa | broad |  |
|  | supracleithrum | absent |  |
|  | ventral humeral ridge | present |  |
| Daeschler et al. 2006 | [anatomical margin and (part_of some (proximal region and (part_of some capitulum of humerus)))] | sharp |  |
|  | [anterior margin and (part_of some ventral humeral ridge)] | distal to | proximal head of humerus |
|  | [muscle scar and (part_of some (proximal region and (part_of some capitulum of humerus)))] | present |  |
|  | [pectoral fin radial bone and (distal_to some ulnare)] | unbranched |  |
|  | [proximal region and (part_of some capitulum of humerus)] | increased size |  |
|  | [ventro-medial region and (part_of some coracoid plate)] | increased size |  |
|  | anal fin | absent |  |
|  | anocleithrum | unornamented |  |
|  | archipterygial fin | absent |  |
|  | basal scute | absent |  |
|  | capitulum of humerus | distal orientation |  |
|  | cleithrum | unornamented |  |
|  | cleithrum | angle |  |
|  | coracoid plate | present |  |
|  | diphycercal tail | present |  |
|  | dorsal fin | absent |  |
|  | ectepicondylar flange | distal to | epiphysis of humerus |
|  | extrascapula | absent |  |
|  | glenoid fossa | decreased elevation | clavicle |
|  | glenoid fossa | lateral orientation |  |
|  | humerus diaphysis | flattened |  |
|  | humerus diaphysis | rectangular |  |
|  | interclavicle | increased size |  |
|  | interclavicle | ornamented |  |
|  | olecranon | absent |  |
|  | paired fin lepidotrichium | absent |  |
|  | proximal head of humerus | strap-shaped |  |
|  | proximal head of humerus | elongated |  |
|  | proximal head of humerus | bifurcated |  |
|  | radius bone | decreased length | humerus |
|  | radius bone | subcylindrical |  |
|  | scapulocoracoid | fused with | cleithrum |
|  | scapulocoracoid | size |  |
|  | scapulocoracoid | triangular |  |
|  | subscapular fossa | present |  |
|  | ulnar condyle | distal orientation |  |
|  | ventral humeral ridge | perpendicular to | humerus cartilage element |
| Ruta 2011 | [adductor crest and (PHENOSCAPE_extends_to some (d573b0-034c-0131-abbd-001ec9b0ea92 and (part_of some femur)))] | absent |  |
|  | [anatomical axis and (part_of some glenoid fossa)] | horizontal |  |
|  | [anatomical margin and (part_of some (clavicle and (continuous with some cleithrum)))] | convex |  |
|  | [anatomical margin and (part_of some (clavicle and (continuous with some cleithrum)))] | straight |  |
|  | [anatomical projection and (part_of some (antero-ventral region and (part_of some cleithrum)))] | present |  |
|  | [anatomical projection and (part_of some (lateral region and (part_of some cleithrum)))] | present |  |
|  | [anatomical projection and (part_of some (posterior margin and (part_of some (flexor surface and (part_of some fibula)))))] | present |  |
|  | [anatomical projection and (part_of some (posterior region and (part_of some cleithrum)))] | absent |  |
|  | [anatomical projection and (part_of some (posterior region and (part_of some tibia)))] | absent |  |
|  | [anatomical projection and (part_of some (ventral region and (part_of some entepicondyle of humerus)))] | absent |  |
|  | [anterior margin and (part_of some cleithrum)] | increased angle to | horizontal plane |
|  | [anterior margin and (part_of some humerus)] | osseous |  |
|  | [anterior margin and (part_of some humerus)] | ridged |  |
|  | [anterior region and (part_of some acetabular part of hip bone)] | smooth |  |
|  | [articular surface and (part_of some distal epiphysis of tibia)] | present |  |
|  | [articular surface and (part_of some distal epiphysis of tibia)] | L-shaped |  |
|  | [articular surface and (part_of some proximal mesomere)] | present |  |
|  | [bone fossa and (part_of some ectepicondyle of humerus)] | absent |  |
|  | [bone fossa and (PHENOSCAPE_serves_as_attachment_site_for some muscle of shoulder)] | present |  |
|  | [clavicle and (has_part some (surface groove and (bearer of some anterolateral orientation)))] | absent |  |
|  | [clavicle and (in_right_side_of some multicellular organism)] | separated from | [clavicle and (in_left_side_of some multicellular organism)] |
|  | [crest and (part_of some (humerus and (adjacent to some entepicondyle of humerus) and (adjacent to some ectepicondyle of humerus)))] | present |  |
|  | [diaphysis and (part_of some cleithrum)] | present |  |
|  | [distal epiphysis of fibula and (bearer of some oblique orientation)] | absent |  |
|  | [distal margin and (part_of some ectepicondylar flange)] | aligned with | ulnar condyle |
|  | [distal region and (part_of some radius bone)] | splayed |  |
|  | [distal tarsal bone and (distal_to some fibulare) and (proximal_to some pedal digit)] | absent |  |
|  | [distal tarsal bone and (distal_to some medial tibial tarsal bone) and (proximal_to some pedal digit)] | absent |  |
|  | [dorsal region and (part_of some cleithrum)] | increased width |  |
|  | [dorsal region and (part_of some cleithrum)] | spatulate |  |
|  | [ectepicondylar flange and (PHENOSCAPE_extends_to some distal epiphysis of humerus)] | present |  |
|  | [humerus diaphysis and (proximal_to some entepicondyle of humerus)] | decreased length | [width and (inheres_in some proximal epiphysis of humerus)] |
|  | [inter-basipterygium joint and (has_part some ischium)] | present |  |
|  | [lateral surface and (part_of some fibula)] | concave |  |
|  | [muscle scar and (proximal_to some radial head of humerus)] | present |  |
|  | [obturator foramen and (part_of some pubis)] | multiple |  |
|  | [ossification center and (part_of some scapula)] | undivided |  |
|  | [posterior margin and (part_of some (entepicondyle of humerus and (bearer of some increased thickness)))] | absent |  |
|  | [posterior margin and (part_of some (ulna and (has_part some (anatomical projection and (bearer of some decreased size) and (bearer of some subcircular)))))] | present |  |
|  | [posterior margin and (part_of some entepicondyle of humerus)] | size | [zone of bone organ and (part_of some (humerus diaphysis and (adjacent to some entepicondyle of humerus)))] |
|  | [posterior region and (part_of some interclavicle)] | increased length | [anterior region and (part_of some interclavicle)] |
|  | [postero-lateral margin and (part_of some (entepicondyle of humerus and (bearer of some concave)))] | absent |  |
|  | [postero-lateral margin and (part_of some entepicondyle of humerus)] | proximal to | radial head of humerus |
|  | [postero-lateral margin and (part_of some entepicondyle of humerus)] | proximal to | ulnar condyle |
|  | [scapulocoracoid and (has_part some (glenoid fossa and (bearer of some subterminal)))] | present |  |
|  | [sulcus and (part_of some (antero-dorsal region and (part_of some (flexor surface and (part_of some fibula)))))] | present |  |
|  | [sulcus and (part_of some (humerus and (adjacent to some entepicondyle of humerus) and (adjacent to some ectepicondyle of humerus)))] | present |  |
|  | [supraglenoid region and (bearer of some bone fossa)] | absent |  |
|  | [surface groove and (part_of some (ventral surface and (part_of some interclavicle)))] | absent |  |
|  | [tubercle and (part_of some (posterior margin and (part_of some (flexor surface and (part_of some fibula)))))] | absent |  |
|  | [ventral margin and (part_of some (postero-dorsal region and (part_of some (ilium and (bearer of some curved)))))] | present |  |
|  | [ventral margin and (part_of some posterodorsal process of ilium)] | acute angle to | [postero-dorsal margin and (part_of some ilium)] |
|  | accessory foramina | present |  |
|  | acetabular part of hip bone | lateral orientation |  |
|  | adductor blade | separated from | diaphysis of femur |
|  | anocleithrum | present |  |
|  | anocleithrum | exposed |  |
|  | anterior distal condyle of femur | increased width | posterior distal condyle of femur |
|  | ascending process of clavicle | present |  |
|  | basal scute | absent |  |
|  | clavicle | [position and (not (flush))] | cleithrum |
|  | clavicle | equilateral triangular |  |
|  | cleithrum | fused with | scapulocoracoid |
|  | cleithrum | unornamented | |
|  | cleithrum | [shape and (not (waisted))] |  |
|  | cnemial crest | distal orientation |  |
|  | cnemial crest | present |  |
|  | condyle of femur | width |  |
|  | coracoid plate | increased size |  |
|  | deltoid process | associated with | dorsal ridge |
|  | deltoid process | present |  |
|  | deltoid process | decreased thickness |  |
|  | distal epiphysis of humerus | aligned with | proximal epiphysis of humerus |
|  | distal epiphysis of ulna | decreased width | proximal epiphysis of ulna |
|  | dorsal iliac process | overlapping | posterodorsal process of ilium |
|  | dorsal iliac process | vertical to | [posterior region and (part_of some iliac neck)] |
|  | dorsal iliac process | splayed |  |
|  | dorsal iliac process | oblique orientation |  |
|  | dorsal iliac process | present |  |
|  | dorsal iliac ridge | present |  |
|  | ectepicondylar flange | present |  |
|  | ectepicondylar foramen | present |  |
|  | entepicondylar foramen | present |  |
|  | entepicondylar foramen | slit-like |  |
|  | entepicondyle of humerus | increased width | [length and (inheres_in some humerus)] |
|  | entepicondyle of humerus | present |  |
|  | entepicondyle of humerus | truncated |  |
|  | fibula | [shape and (not (waisted))] |  |
|  | fibula | [width and (PATO_increased_in_magnitude_relative_to some (length and (inheres_in some fibula)))] |  |
|  | fourth trochanter | rugose |  |
|  | glenoid fossa | ventral to | clavicle |
|  | glenoid fossa | lateral orientation |  |
|  | head of femur | increased size |  |
|  | hindlimb interepipodial space | absent |  |
|  | humerus | decreased length | trunk vertebra |
|  | humerus | [length and (PATO_decreased_in_magnitude_relative_to some (width and (inheres_in some humerus)))] |  |
|  | humerus | dorso-ventrally flattened |  |
|  | humerus diaphysis | [shape and (not (waisted))] |  |
|  | infraglenoid buttress | absent |  |
|  | interclavicle | unornamented |  |
|  | interclavicle | increased size |  |
|  | interclavicle | rhomboid |  |
|  | interclavicle | [width and (PATO_increased_in_magnitude_relative_to some (length and (inheres_in some interclavicle)))] |  |
|  | intercondylar fossa | decreased length | [distal epiphysis of femur and (bearer of some width)] |
|  | intercondylar fossa | present |  |
|  | intermedium (fore) | decreased length | ulna |
|  | intermedium (fore) | present |  |
|  | intermedium (fore) | bulbous |  |
|  | internal trochanter | separated from | diaphysis of femur |
|  | internal trochanter | present |  |
|  | latissimus dorsi process | offset | ectepicondyle of humerus |
|  | latissimus dorsi process | in contact with | deltopectoral crest |
|  | latissimus dorsi process | bulbous |  |
|  | manual digit digitopodial skeleton | count |  |
|  | mesial pelvic ridge | offset | iliac neck |
|  | mesomere 4 | absent |  |
|  | mesomere 5 | absent |  |
|  | metacarpal bone | decreased length | radius bone |
|  | metacarpal bone | present |  |
|  | metacarpal bone | length |  |
|  | metacarpal bone | length |  |
|  | metatarsal bone | decreased length | tibia |
|  | metatarsal bone | length |  |
|  | metatarsal bone | length |  |
|  | metatarsal bone | present |  |
|  | neck of humerus | absent |  |
|  | olecranon | absent |  |
|  | paired fin radial bone | absent |  |
|  | parasternal process | [shape and (not (parallel-sided))] |  |
|  | pectoral fin lepidotrichium | absent |  |
|  | pectoral process of humerus | triangular |  |
|  | pectoral process of humerus | present |  |
|  | pectoral process of humerus | increased thickness |  |
|  | pedal digit digitopodial skeleton | count |  |
|  | pelvic fin lepidotrichium | absent |  |
|  | pelvic girdle skeleton | [structure and (not (sutured to))] |  |
|  | postaxial process of the fibula | absent |  |
|  | postbranchial lamina | present |  |
|  | posterodorsal process of ilium | [length and (PATO_decreased_in_magnitude_relative_to some (width and (inheres_in some posterodorsal process of ilium)))] |  |
|  | process 2 | present |  |
|  | process 3 | present |  |
|  | process 4 | present |  |
|  | proximal epiphysis of metacarpal bone | symmetrical |  |
|  | proximal epiphysis of metatarsal bone | symmetrical |  |
|  | proximal epiphysis of radius | decreased size |  |
|  | proximal head of humerus | bifurcated |  |
|  | proximal head of humerus | elongated |  |
|  | proximal head of humerus | strap-shaped |  |
|  | proximal tarsal bone | absent |  |
|  | proximal tarsal bone | absent |  |
|  | radial head of humerus | terminal |  |
|  | radius bone | increased length | ulna |
|  | radius bone | decreased length | humerus |
|  | radius bone | [length and (PATO_increased_in_magnitude_relative_to some (width and (inheres_in some (d573b0-034c-0131-abbd-001ec9b0ea92 and (part_of some radius bone)))))] |  |
|  | radius bone | present |  |
|  | sacral rib | increased width | presacral rib |
|  | sacral rib | decreased width | trunk rib |
|  | scapular blade | present |  |
|  | scapular blade | decreased size |  |
|  | scapulocoracoid | exposed |  |
|  | subscapular fossa | present |  |
|  | supinator process | absent |  |
|  | supraacetabular buttress | decreased size | postacetabular buttress |
|  | supracleithrum | absent |  |
|  | supraglenoid foramen | size |  |
|  | supraglenoid foramen | present |  |
|  | supraglenoid foramen | subcircular |  |
|  | tibia | increased length | femur |
|  | tibia | [shape and (not (waisted))] |  |
|  | tibia | [width and (PATO_increased_in_magnitude_relative_to some (length and (inheres_in some tibia)))] |  |
|  | transverse pelvic ridge | absent |  |
|  | ulna | [width and (PATO_similar_in_magnitude_relative_to some (length and (inheres_in some ulna)))] |  |
|  | ulnar facet of the humerus | separated from | radial facet |
|  | ventral humeral ridge | separated from | [postero-medial margin and (part_of some entepicondyle of humerus)] |
|  | ventral humeral ridge | present |  |
|  | ventral humeral ridge | transverse orientation |  |
| Swartz 2012 | [anatomical conduit and (part_of some iliac neck)] | absent |  |
|  | [anatomical margin and (part_of some cleithrum)] | concave |  |
|  | [anatomical region and (adjacent to some (facet and (part_of some radius bone)))] | increased size |  |
|  | [anatomical region and (adjacent to some (facet and (part_of some radius bone)))] | sharp |  |
|  | [anterior margin and (part_of some ventral humeral ridge)] | distal to | proximal epiphysis of humerus |
|  | [articulation and (part_of some (anterior side and (part_of some anocleithrum)))] | absent |  |
|  | [distal region and (part_of some body of rib)] | increased width |  |
|  | [extratemporal bone and (posterior_to some spiracle)] | absent |  |
|  | [facet and (part_of some radius bone)] | distal orientation |  |
|  | [facet and (part_of some ulna)] | distal orientation |  |
|  | [muscle scar and (part_of some (facet and (part_of some radius bone)))] | present |  |
|  | [pectoral fin radial bone and (distal_to some ulnare)] | unbranched |  |
|  | [posterior margin and (part_of some tabular bone)] | posterior to | [posterior margin and (part_of some interparietal bone)] |
|  | anal fin | absent |  |
|  | anocleithrum | exposed |  |
|  | anocleithrum | teardrop-shaped |  |
|  | archipterygial fin | absent |  |
|  | basicranial fenestra | absent |  |
|  | body of rib | increased length | [bony vertebral centrum and (has_part some diameter)] |
|  | body of rib | straight |  |
|  | cleithrum | [orientation and (not (vertical))] |  |
|  | cleithrum | unornamented |  |
|  | coracoid foramen | absent |  |
|  | coracoid plate | increased size |  |
|  | deltopectoral crest | present |  |
|  | dermal intracranial joint | absent |  |
|  | diphycercal tail | present |  |
|  | dorsal fin | absent |  |
|  | ectepicondylar flange | distal to | condyle of humerus |
|  | entepicondyle of humerus | square |  |
|  | entepicondyle of humerus | parallelogram |  |
|  | extrascapula | absent |  |
|  | glenoid fossa | parallel to | clavicle |
|  | glenoid fossa | lateral orientation |  |
|  | glenoid fossa | [height and (PATO_decreased_in_magnitude_relative_to some (width and (inheres_in some glenoid fossa)))] |  |
|  | humerus diaphysis | flattened |  |
|  | humerus diaphysis | rectangular |  |
|  | individual digit of digitopodial skeleton | present |  |
|  | interclavicle | diamond shaped |  |
|  | interclavicle | increased size |  |
|  | interclavicle | exposed |  |
|  | interclavicle | ornamented |  |
|  | olecranon | absent |  |
|  | opercle | absent |  |
|  | pectoral fin lepidotrichium | absent |  |
|  | pelvic fin lepidotrichium | absent |  |
|  | postaxial process of the fibula | absent |  |
|  | postbranchial lamina | present |  |
|  | posterior process of ilium | posterodorsal orientation | ilium |
|  | posttemporal | absent |  |
|  | preopercle | present |  |
|  | preopercle | decreased size |  |
|  | proximal head of humerus | convex |  |
|  | radius bone | decreased length | humerus |
|  | radius bone | increased length | ulna |
|  | radius bone | subcylindrical |  |
|  | rib | [structure and (not (overlapping))] |  |
|  | rib | increased size |  |
|  | scapular blade | absent |  |
|  | scapulocoracoid | platelike |  |
|  | scapulocoracoid | increased size |  |
|  | spiracular notch | increased width |  |
|  | supinator process | present |  |
|  | supracleithrum | absent |  |
|  | supraneural bone | absent |  |
|  | supratemporal bone | separated from | interparietal bone |
| Vallin and Laurin 2004 | [anatomical projection and (part_of some (dorsal region and (part_of some iliac blade)))] | present |  |
|  | [anatomical projection and (part_of some (posterior region and (part_of some iliac blade)))] | present |  |
|  | [dorsal region and (part_of some cleithrum)] | increased size |  |
|  | [ossification center and (part_of some pelvic girdle skeleton)] | count |  |
|  | [ossification center and (part_of some scapulocoracoid)] | count |  |
|  | adductor blade | elongated |  |
|  | anocleithrum | present |  |
|  | anterior humeral ridge | present |  |
|  | clavicle | present |  |
|  | clavicle blade | present |  |
|  | ectepicondylar foramen | present |  |
|  | ectepicondyle of humerus | increased height |  |
|  | entepicondylar foramen | present |  |
|  | entepicondyle of humerus | increased width |  |
|  | humerus | decreased length | [length and (inheres_in some thoracic vertebra)] |
|  | humerus | present |  |
|  | lateral extrascapular | absent |  |
|  | manual digit digitopodial skeleton | count |  |
|  | median extrascapular | absent |  |
|  | olecranon | absent |  |
|  | parasternal process | absent |  |
|  | postbranchial lamina | present |  |
|  | radius bone | separated from | ulna |
|  | scapulocoracoid | fused with | cleithrum |
|  | supinator process | decreased size |  |
|  | ulna | decreased length | radius bone |

**EQs from Coates 1996 for *Acanthostega***

| Entities | Qualities | Related Entities | |
| --- | --- | --- | --- |
| interclavicle | medial to | pectoral girdle skeleton | |
| scapulocoracoid | decreased thickness |  | |
| scapulocoracoid | broad |  | |
| clavicle | present |  | |
| anocleithrum | present |  | |
| cleithrum | increased height |  | |
| [suture and (part_of some coracoid bone)] | absent |  | |
| [growth line and (part_of some coracoid bone)] | absent |  | |
| glenoid fossa | increased length | [proximal head of humerus and (bearer of some width)] | |
| glenoid fossa | coiled |  | |
| glenoid fossa | strap-shaped |  | |
| glenoid fossa | posterolateral orientation |  | |
| glenoid fossa | concave |  | |
| coracoid bone | decreased thickness |  | |
| coracoid bone | in contact with | clavicle | |
| [glenoid region and (part_of some coracoid bone)] | curved medial |  | |
| [anatomical conduit and (part_of some (antero-ventral region and (part_of some glenoid region)))] | count |  | |
| [anatomical conduit and (anterior_to some glenoid region)] | count |  | |
| [scapulocoracoid and (anterior_to some glenoid region)] | decreased thickness |  | |
| [anatomical conduit and (part_of some (scapulocoracoid and (bearer of some spatial pattern)))] | triangular |  | |
| [foramen C and (adjacent to some cleithrum)] | present |  | |
| [postero-dorsal region and (part_of some supraglenoid buttress)] | smooth |  | |
| supraglenoid buttress | ossified |  | |
| [postero-dorsal region and (part_of some supraglenoid buttress)] | triangular |  | |
| supraglenoid buttress | in contact with |  | |
| infraglenoid buttress | absent |  | |
| [medial surface and (part_of some glenoid region)] | increased thickness |  | |
| [scapula and (UBERON_attaches_to some cleithrum)] | increased thickness |  | |
| [crest and (part_of some (antero-dorsal region and (part_of some scapula)))] | increased thickness |  | |
| scapula | decreased thickness |  | |
| [anterior side and (part_of some surface groove)] | adjacent to | [crest and (part_of some (antero-dorsal region and (part_of some scapula)))] | |
| [surface groove and (part_of some dorsal clavicular process)] | present |  |  |
| [scapula and (continuous with some cleithrum)] | anterior to |  |  |
| scapula | fused with | cleithrum | |
| scapular blade | decreased size |  | |
| [lower region and (part_of some cleithrum)] | surrounding | scapular blade | |
| [intramembranous bone tissue and (part_of some cleithrum) and (part_of some scapular blade)] | surrounding | trabecular bone tissue | |
| [dorsal region and (part_of some (pectoral girdle skeleton and (has_part some dermal bone)))] | overlapping | [endochondral bone and (part_of some pectoral girdle skeleton)] | |
| [ventral region and (part_of some (pectoral girdle skeleton and (has_part some dermal bone)))] | present |  | |
| anteroventral process of cleithrum | present |  | |
| [dorsal region and (part_of some cleithrum)] | increased size |  | |
| postbranchial lamina | present |  | |
| cleithrum | increased height |  | |
| [muscle scar and (part_of some cleithrum)] | inconspicuous |  | |
| cleithrum | unornamented |  | |
| [muscle scar and (part_of some cleithrum)] | dorsal to | foramen C | |
| anteroventral process of cleithrum | overlapped by | [posterior region and (part_of some ascending process of clavicle)] | |
| anteroventral process of cleithrum | increased size |  | |
| postbranchial lamina | [structure and (not (overlapping))] | clavicle | |
| anocleithrum | unornamented |  | |
| [postero-ventral region and (part_of some anocleithrum)] | overlapped by | cleithrum | |
| anocleithrum | anterodorsal orientation | [distal region and (part_of some basicranium)] | |
| [antero-dorsal region and (part_of some anocleithrum)] | raised |  | |
| [posterior margin and (part_of some (antero-dorsal region and (part_of some anocleithrum)))] | perforate |  | |
| [posterior margin and (part_of some anocleithrum)] | serrated |  | |
| anocleithrum | [position and (not (medial orientation))] |  | |
| ascending process of clavicle | cylindrical |  | |
| clavicle blade | subtriangular |  | |
| clavicle blade | subcircular |  | |
| clavicle blade | broad |  | |
| [ventral surface and (part_of some clavicle)] | ornamentation |  | |
| dorsal clavicular process | recurved |  | |
| dorsal clavicular process | grooved |  | |
| [crest and (part_of some (posterior region and (part_of some dorsal clavicular process)))] | present |  | |
| [lamina and (part_of some (posterior region and (part_of some dorsal clavicular process)))] | increased size |  | |
| interclavicle | diamond shaped |  | |
| interclavicle | ornamented |  | |
| parasternal process | increased size |  | |
| parasternal process | increased width |  | |
| humerus | dorso-ventrally flattened |  | |
| humerus | L-shaped |  | |
| entepicondyle of humerus | increased size |  | |
| ectepicondylar flange | increased size |  | |
| [articular surface and (part_of some proximal epiphysis of humerus)] | acute angle to | condyle of humerus | |
| proximal head of humerus | elliptic |  |  |
| [scapulohumeralis muscle and (UBERON_has_muscle_insertion some humerus)] | present |  |  |
| [humerus and (bearer of some (e00-02f8-0132-ee8e-005056010073 and (has_part some muscle scar)))] | present |  |  |
| latissimus dorsi process | increased size |  | |
| latissimus dorsi process | elongated |  | |
| latissimus dorsi process | perforate |  | |
| process 2 | adjacent to | entepicondylar foramen | |
| [anatomical surface and (posterior_to some latissimus dorsi process)] | smooth |  | |
| supinator process | aligned with | latissimus dorsi process | |
| supinator process | decreased height |  | |
| supinator process | subcircular |  | |
| [crest and (UBERON_anteriorly_connected_to some supinator process) and (UBERON_posteriorly_connected_to some ectepicondyle of humerus)] | overlapping | ectepicondylar foramen | |
| [crest and (UBERON_anteriorly_connected_to some supinator process) and (UBERON_posteriorly_connected_to some ectepicondyle of humerus)] | decreased height |  | |
| [medial margin and (part_of some entepicondyle of humerus)] | bifurcated |  | |
| process 3 | present |  | |
| process 4 | present |  | |
| [bone foramen and (part_of some entepicondyle of humerus)] | count |  | |
| radial head of humerus | separated from | ulnar condyle | |
| radial head of humerus | terminal |  | |
| radial head of humerus | anterior orientation |  | |
| ulnar condyle | anterior orientation |  | |
| ulnar condyle | distal to | ectepicondyle of humerus | |
| ulnar condyle | subcircular |  | |
| ulnar condyle | lateral orientation |  | |
| [surface groove and (adjacent to some ulnar condyle)] | separating | ectepicondyle of humerus | |
| radial head of humerus | split |  | |
| radial head of humerus | strap-shaped |  | |
| proximal epiphysis of humerus | convex |  | |
| proximal epiphysis of humerus | keel-shaped |  | |
| deltopectoral crest | extends to | proximal epiphysis of humerus | |
| deltopectoral crest | vertical |  | |
| [anterior region and (part_of some (ventral surface and (part_of some humerus)))] | smooth |  | |
| [posterior region and (part_of some (ventral surface and (part_of some humerus)))] | concave |  | |
| [ventral surface and (part_of some humerus)] | split |  | |
| [distal region and (part_of some (ventral surface and (part_of some humerus)))] | smooth |  | |
| [surface groove and (PHENOSCAPE_extends_from some epipodial facet)] | decreased depth |  | |
| [surface groove and (PHENOSCAPE_extends_from some epipodial facet)] | broad |  | |
| ventral humeral ridge | in contact with | [medial region and (part_of some entepicondyle of humerus)] | |
| ventral humeral ridge | decreased height |  | |
| ventral humeral ridge | subcircular |  | |
| [bone foramen and (in_distal_side_of some ventral humeral ridge)] | present |  | |
| ulna | dorso-ventrally flattened |  | |
| ulna | decreased length | radius bone | |
| diaphysis of ulna | absent |  | |
| [distal region and (part_of some (dorsal surface and (part_of some ulna)))] | decreased width |  | |
| [proximal region and (part_of some (dorsal surface and (part_of some ulna)))] | increased width |  | |
| [posterior margin and (part_of some (dorsal surface and (part_of some ulna)))] | convex |  | |
| [anterior margin and (part_of some (dorsal surface and (part_of some ulna)))] | straight |  | |
| olecranon | absent |  | |
| [distal surface and (part_of some ulna)] | notched |  | |
| [articular surface and (part_of some ulna)] | offset | [posterior region and (part_of some (distal surface and (part_of some ulna)))] | |
| [distal region and (part_of some radius bone)] | flattened |  | |
| radius bone | spatulate |  | |
| radius bone | subcylindrical |  | |
| [dorsal surface and (part_of some radius bone)] | increased size |  | |
| [distal surface and (part_of some radius bone)] | concave |  | |
| [ventral surface and (part_of some radius bone)] | concave |  | |
| [crest and (part_of some (antero-ventral region and (part_of some radius bone)))] | elongated |  | |
| [posterior surface and (part_of some radius bone)] | smooth |  | |
| [posterior surface and (part_of some radius bone)] | cut |  | |
| intermedium (fore) | ossified |  | |
| [distal region and (part_of some intermedium (fore))] | increased size |  | |
| intermedium (fore) | subcylindrical |  | |
| manual digit 1 digitopodial skeleton | count |  | |
| manual digit 2 digitopodial skeleton | count |  | |
| manual digit 3 digitopodial skeleton | count |  | |
| manual digit 4 digitopodial skeleton | count |  | |
| manual digit 5 digitopodial skeleton | count |  | |
| manual digit 6 digitopodial skeleton | count |  | |
| manual digit 7 digitopodial skeleton | count |  | |
| manual digit 8 digitopodial skeleton | count |  | |
| manual digit 1 digitopodial skeleton | decreased length |  | |
| manual digit 2 digitopodial skeleton | decreased length |  | |
| manual digit 3 digitopodial skeleton | decreased length |  | |
| manual digit 8 digitopodial skeleton | slender |  | |
| phalanx of manus | hourglass-shaped |  | |
| middle phalanx of manual digit 3 | increased length | middle phalanx of manual digit 2 | |
| middle phalanx of manual digit 3 | increased length | middle phalanx of manual digit 1 | |
| [suture and (part_of some pelvic girdle skeleton)] | absent |  | |
| [dorsal region and (part_of some ilium)] | bifurcated |  | |
| iliac neck | decreased width |  | |
| iliac neck | posterior to | supraacetabular buttress | |
| iliac neck | dorsal to | [posterior region and (part_of some acetabular part of hip bone)] | |
| iliac neck | imperforate |  | |
| posterior process of ilium | spatulate |  | |
| [dorsal region and (part_of some dorsal iliac process)] | increased thickness |  | |
| dorsal iliac process | bulbous |  | |
| posterior process of ilium | lateral to | dorsal iliac process | |
| [distal region and (part_of some posterior process of ilium)] | decreased thickness |  | |
| iliac blade | smooth |  | |
| [proximal region and (part_of some dorsal iliac process)] | striated |  | |
| [anatomical projection and (part_of some iliac neck)] | transverse orientation |  | |
| [anatomical projection and (part_of some iliac neck)] | U-shaped |  | |
| puboischiadic plate | elongated |  | |
| acetabular part of hip bone | anterior to | dorsal iliac process | |
| supraacetabular buttress | separated from | iliac neck | |
| supraacetabular buttress | decreased size | postacetabular buttress | |
| acetabular part of hip bone | subtriangular |  | |
| [ventral margin and (part_of some acetabular labrum)] | in contact with | postacetabular buttress | |
| [ventral margin and (part_of some acetabular labrum)] | increased thickness |  | |
| [lateral surface and (part_of some (puboischiadic plate and (ventral_to some acetabular labrum)))] | perforate |  | |
| [bone foramen and (in_anterior_side_of some puboischiadic plate)] | count |  | |
| [bone foramen and (part_of some puboischiadic plate)] | increased size |  | |
| [bone foramen and (part_of some puboischiadic plate)] | anteroventral to | postacetabular buttress | |
| puboischiadic plate | posterior to | acetabular part of hip bone | |
| acetabular part of hip bone | decreased thickness |  | |
| [dorsal margin and (part_of some acetabular rim)] | [thickness and (not (increased thickness))] |  | |
| [medial surface and (part_of some puboischiadic plate)] | smooth |  | |
| [anatomical projection and (part_of some (medial surface and (part_of some puboischiadic plate)))] | broad |  | |
| bone foramen | anterior to | [anatomical projection and (part_of some (medial surface and (part_of some puboischiadic plate)))] | |
| [bone foramen and (part_of some (dorsal region and (part_of some pubis)))] | in contact with | [bone foramen and (part_of some (lateral region and (part_of some puboischiadic plate)))] | |
| [bone foramen and (part_of some pubis)] | count |  | |
| [anatomical projection and (part_of some pubic symphysis)] | increased thickness |  | |
| femur | increased length | humerus | |
| [muscle scar and (part_of some (ventral surface and (part_of some femur)))] | distal to | proximal epiphysis of femur | |
| trochanter | distal to | proximal epiphysis of femur | |
| fourth trochanter | separated from | internal trochanter | |
| femur | torsioned |  | |
| intercondylar fossa | anterior orientation |  | |
| adductor blade | elongated |  | |
| adductor crest | decreased length |  | |
| [articular surface and (part_of some (distal epiphysis of femur and (part_of some ventral surface)))] | present |  | |
| [articular surface and (part_of some (proximal epiphysis of femur and (part_of some ventral surface)))] | present |  | |
| [articular surface and (part_of some proximal epiphysis of femur)] | broad |  | |
| [posterior region and (part_of some (articular surface and (part_of some proximal epiphysis of femur)))] | distal to | [anterior region and (part_of some (articular surface and (part_of some proximal epiphysis of femur)))] | |
| [intertrochanteric fossa and (UBERON_encloses some bone foramen)] | count |  | |
| intertrochanteric fossa | smooth |  | |
| [articular surface and (part_of some proximal epiphysis of femur)] | separated from | internal trochanter | |
| proximal epiphysis of femur | ossified |  | |
| head of femur | [width and (PATO_decreased_in_magnitude_relative_to some (length and (inheres_in some head of femur)))] |  | |
| diaphysis of femur | increased length |  | |
| [a445b30-0390-0132-2b01-005056010074 and (part_of some femur)] | convex |  | |
| [bone fossa and (part_of some (posterior margin and (part_of some (a445b30-0390-0132-2b01-005056010074 and (part_of some femur)))))] | present |  | |
| [a445b30-0390-0132-2b01-005056010074 and (part_of some femur)] | smooth |  | |
| lateral condyle of femur | increased height | anterior distal condyle of femur | |
| [intercondylar fossa and (UBERON_encloses some bone foramen)] | count |  | |
| [articular surface and (part_of some proximal epiphysis of femur)] | ossified |  | |
| [anterior distal condyle of femur and (has_part some tibial facet of femur)] | present |  | |
| [lateral condyle of femur and (has_part some fibula facet of femur)] | present |  | |
| intercondylar fossa | anterior orientation |  | |
| popliteal area | ventral orientation |  | |
| fibula facet of femur | absent |  | |
| fibula facet of femur | anteroventral orientation |  | |
| tibial facet of femur | posterolateral orientation |  | |
| adductor blade | increased size |  | |
| adductor blade | aligned with | condyle of femur | |
| adductor blade | extends beyond | [d573b0-034c-0131-abbd-001ec9b0ea92 and (part_of some femur)] | |
| internal trochanter | separated from | head of femur | |
| [pit and (part_of some internal trochanter)] | present |  | |
| adductor blade | distal to | internal trochanter | |
| crest | proximal to | [posterior margin and (part_of some adductor blade)] | |
| [proximal region and (part_of some adductor blade)] | smooth |  | |
| [distal region and (part_of some adductor blade)] | furrowed |  | |
| [distal region and (part_of some fourth trochanter)] | foveate |  | |
| adductor crest | acute |  | |
| adductor crest | decreased length |  | |
| adductor crest | separated from | tibial facet of femur | |
| tibia | increased width |  | |
| tibia | increased length | fibula | |
| tibia | decreased length | femur | |
| cnemial crest | subcircular |  | |
| cnemial crest | increased size |  | |
| cnemial crest | smooth |  | |
| [anterior margin and (part_of some tibia)] | straight |  | |
| [posterior margin and (part_of some tibia)] | straight |  | |
| tibia | [shape and (not (torsioned))] |  | |
| diaphysis of tibia | absent |  | |
| hindlimb interepipodial space | absent |  | |
| [bone foramen and (part_of some (ventral surface and (part_of some tibia)))] | present |  | |
| [bone fossa and (part_of some (distal region and (part_of some (ventral surface and (part_of some tibia)))))] | present |  | |
| [anatomical projection and (part_of some (ventral surface and (part_of some tibia)))] | absent |  | |
| [lateral margin and (part_of some tibia)] | decreased length | [medial margin and (part_of some tibia)] | |
| [lateral margin and (part_of some tibia)] | decreased width | [medial margin and (part_of some tibia)] | |
| [lateral margin and (part_of some tibia)] | overlapped by | [medial margin and (part_of some fibula)] | |
| [bone fossa and (in_anterior_side_of some (medial margin and (part_of some tibia)))] | present |  | |
| [articular surface and (part_of some distal epiphysis of tibia)] | L-shaped |  | |
| [articular surface and (part_of some distal epiphysis of tibia)] | bifurcated |  | |
| [anterior margin and (part_of some fibula)] | concave |  | |
| [posterior margin and (part_of some fibula)] | concave |  | |
| fibula | dorso-ventrally flattened |  | |
| fibula | broad |  | |
| fibula | waisted |  | |
| [anatomical projection and (part_of some (ventral surface and (part_of some (posterior region and (part_of some fibula)))))] | flattened |  | |
| [bone fossa and (part_of some (posterior margin and (part_of some fibula)))] | present |  | |
| [surface groove and (in_anterior_side_of some (ventral surface and (part_of some fibula)))] | present |  | |
| fibula | smooth |  | |
| proximal epiphysis of fibula | linear |  | |
| proximal epiphysis of fibula | decreased width |  | |
| [distal surface and (part_of some proximal epiphysis of fibula)] | convex |  | |
| fibula | torsioned |  | |
| pedal digit 1 digitopodial skeleton | present |  | |
| pedal digit 2 digitopodial skeleton | present |  | |
| pedal digit 3 digitopodial skeleton | present |  | |
| pedal digit 4 digitopodial skeleton | present |  | |
| pedal digit 5 digitopodial skeleton | present |  | |
| pedal digit 6 digitopodial skeleton | present |  | |
| pedal digit 7 digitopodial skeleton | present |  | |
| pedal digit 8 digitopodial skeleton | present |  | |
| tarsal skeleton | ossified |  | |
| carpal skeleton | poorly ossified |  | |
| medial tibial tarsal bone | rectangular |  | |
| fibulare | 2-D shape |  | |
| hindlimb intermedium | ovate |  | |
| hindlimb intermedium | decreased size |  | |
| fibulare | decreased size |  | |
| fibulare | in contact with | digit 6 digitopodial skeleton | |
| fibulare | in contact with | digit 8 digitopodial skeleton | |
| fibulare | in contact with | pedal digit 7 digitopodial skeleton | |
| navicular bone of pes | absent |  | |
| distal tarsal bone | increased size |  | |
| distal tarsal bone | in contact with | pedal digit 2 digitopodial skeleton | |
| distal tarsal bone | in contact with | pedal digit 3 digitopodial skeleton | |
| distal tarsal bone | in contact with | pedal digit 4 digitopodial skeleton | |
| distal tarsal bone | in contact with | pedal digit 5 digitopodial skeleton | |
| distal tarsal bone | dorso-ventrally flattened |  | |
| distal tarsal bone | subrectangular |  | |
| [distal tarsal bone and (adjacent to some pedal digit 5 digitopodial skeleton)] | notched |  | |
| pedal digit digitopodial skeleton | increased size | manual digit digitopodial skeleton | |
| [distal region and (part_of some pedal digit 2 digitopodial skeleton)] | tapered |  | |
| [proximal region and (part_of some pedal digit 2 digitopodial skeleton)] | broad |  | |
| pedal digit 1 digitopodial skeleton | count |  | |
| pedal digit 2 digitopodial skeleton | count |  | |
| pedal digit 3 digitopodial skeleton | count |  | |
| pedal digit 4 digitopodial skeleton | count |  | |
| pedal digit 5 digitopodial skeleton | count |  | |
| pedal digit 6 digitopodial skeleton | count |  | |
| pedal digit 7 digitopodial skeleton | count |  | |
| pedal digit 8 digitopodial skeleton | count |  | |

**EQs from Matrices for *Barameda***

|  | Entities | Qualities | Related Entities |
| --- | --- | --- | --- |
| Ruta 2011 | [anatomical projection and (part_of some (posterior region and (part_of some cleithrum)))] | present |  |
|  | [anatomical projection and (part_of some (ventral region and (part_of some entepicondyle of humerus)))] | absent |  |
|  | [anterior margin and (part_of some cleithrum)] | decreased angle to | horizontal plane |
|  | [anterior margin and (part_of some humerus)] | convex |  |
|  | [anterior margin and (part_of some humerus)] | osseous |  |
|  | [articular surface and (part_of some proximal mesomere)] | present |  |
|  | [basal region and (part_of some pectoral fin lepidotrichium)] | increased length |  |
|  | [bone fossa and (part_of some ectepicondyle of humerus)] | present |  |
|  | [bone fossa and (PHENOSCAPE_serves_as_attachment_site_for some muscle of shoulder)] | present |  |
|  | [cleithrum and (bearer of some waisted)] | present |  |
|  | [cleithrum and (continuous with some clavicle)] | [shape and (not (e281da0-3fc2-0132-befb-005056010073))] |  |
|  | [crest and (part_of some (humerus and (adjacent to some entepicondyle of humerus) and (adjacent to some ectepicondyle of humerus)))] | absent |  |
|  | [distal margin and (part_of some ectepicondylar flange)] | aligned with | ulnar condyle |
|  | [distal region and (part_of some radius bone)] | blade-like |  |
|  | [dorsal region and (part_of some deltoid process)] | bulbous |  |
|  | [ectepicondylar flange and (PHENOSCAPE_extends_to some distal epiphysis of humerus)] | absent |  |
|  | [humerus diaphysis and (proximal_to some entepicondyle of humerus)] | decreased length | [width and (inheres_in some proximal epiphysis of humerus)] |
|  | [muscle scar and (proximal_to some radial head of humerus)] | present |  |
|  | [paired fin radial bone and (has_part some skeletal joint)] | present |  |
|  | [posterior margin and (part_of some (entepicondyle of humerus and (bearer of some increased thickness)))] | absent |  |
|  | [posterior margin and (part_of some (ulna and (has_part some (anatomical projection and (bearer of some decreased size) and (bearer of some subcircular)))))] | present |  |
|  | [posterior margin and (part_of some entepicondyle of humerus)] | size | [zone of bone organ and (part_of some (humerus diaphysis and (adjacent to some entepicondyle of humerus)))] |
|  | [postero-lateral margin and (part_of some (entepicondyle of humerus and (bearer of some concave)))] | absent |  |
|  | [postero-lateral margin and (part_of some entepicondyle of humerus)] | distal to | radial head of humerus |
|  | [postero-lateral margin and (part_of some entepicondyle of humerus)] | distal to | ulnar condyle |
|  | [scapulocoracoid and (has_part some (attachment site and (bearer of some shape)))] | tripodal |  |
|  | [sulcus and (part_of some (humerus and (adjacent to some entepicondyle of humerus) and (adjacent to some ectepicondyle of humerus)))] | absent |  |
|  | ascending process of clavicle | present |  |
|  | basal scute | absent |  |
|  | cleithrum | attached to | scapulocoracoid |
|  | cleithrum | ornamented |  |
|  | coracoid plate | decreased size |  |
|  | deltoid process | present |  |
|  | deltoid process | subcircular |  |
|  | distal epiphysis of humerus | aligned with | proximal epiphysis of humerus |
|  | distal epiphysis of ulna | increased width | proximal epiphysis of ulna |
|  | ectepicondylar flange | present |  |
|  | entepicondylar foramen | elliptic |  |
|  | entepicondylar foramen | present |  |
|  | entepicondylar foramen | subcircular |  |
|  | entepicondyle of humerus | increased width | [length and (inheres_in some humerus)] |
|  | entepicondyle of humerus | present |  |
|  | entepicondyle of humerus | blunt |  |
|  | entepicondyle of humerus | subcircular |  |
|  | glenoid fossa | dorsal to | clavicle |
|  | glenoid fossa | posterior orientation |  |
|  | glenoid fossa | ventral orientation |  |
|  | humerus | [shape and (not (dorso-ventrally flattened))] |  |
|  | humerus | [length and (PATO_decreased_in_magnitude_relative_to some (width and (inheres_in some humerus)))] |  |
|  | humerus diaphysis | waisted |  |
|  | interclavicle | decreased size |  |
|  | interclavicle | scute-like |  |
|  | intermedium (fore) | present |  |
|  | intermedium (fore) | hourglass-shaped |  |
|  | intermedium (fore) | waisted |  |
|  | latissimus dorsi process | absent |  |
|  | manual digit digitopodial skeleton | absent |  |
|  | metacarpal bone | absent |  |
|  | neck of humerus | present |  |
|  | olecranon | absent |  |
|  | paired fin radial bone | present |  |
|  | pectoral fin radial bone | bifurcated |  |
|  | pectoral process of humerus | present |  |
|  | pectoral process of humerus | triangular |  |
|  | pectoral process of humerus | decreased thickness |  |
|  | postaxial process of the ulnare | absent |  |
|  | process 2 | absent |  |
|  | process 3 | absent |  |
|  | process 4 | absent |  |
|  | proximal epiphysis of radius | decreased size |  |
|  | proximal head of humerus | subcircular |  |
|  | radial head of humerus | terminal |  |
|  | radius bone | increased length | ulna |
|  | radius bone | increased length | humerus |
|  | radius bone | present |  |
|  | radius bone | [length and (PATO_decreased_in_magnitude_relative_to some (width and (inheres_in some (d573b0-034c-0131-abbd-001ec9b0ea92 and (part_of some radius bone)))))] |  |
|  | scapular blade | absent |  |
|  | subscapular fossa | absent |  |
|  | ulna | [width and (PATO_increased_in_magnitude_relative_to some (length and (inheres_in some ulna)))] |  |
|  | ulnar facet of the humerus | undivided | radial facet |
|  | ventral humeral ridge | extends to | [postero-medial margin and (part_of some entepicondyle of humerus)] |
|  | ventral humeral ridge | present |  |
|  | ventral humeral ridge | oblique orientation |  |
| Swartz 2012 | [anatomical margin and (part_of some cleithrum)] | convex |  |
|  | [anatomical margin and (part_of some cleithrum)] | straight |  |
|  | [anatomical region and (adjacent to some (facet and (part_of some radius bone)))] | cylindrical |  |
|  | [anatomical region and (adjacent to some (facet and (part_of some radius bone)))] | decreased height |  |
|  | [anterior margin and (part_of some median extrascapular)] | decreased length |  |
|  | [anterior margin and (part_of some ventral humeral ridge)] | adjacent to | proximal epiphysis of humerus |
|  | [basal region and (part_of some paired fin lepidotrichium)] | elongated |  |
|  | [extratemporal bone and (posterior_to some spiracle)] | in contact with | supratemporal bone |
|  | [extratemporal bone and (posterior_to some spiracle)] | anterior orientation |  |
|  | [extratemporal bone and (posterior_to some spiracle)] | present |  |
|  | [facet and (part_of some radius bone)] | distal orientation |  |
|  | [facet and (part_of some ulna)] | distal orientation |  |
|  | [muscle scar and (part_of some (facet and (part_of some radius bone)))] | absent |  |
|  | [pectoral fin radial bone and (continuous with some ulnare)] | present |  |
|  | [posterior margin and (part_of some tabular bone)] | anterior to | [posterior margin and (part_of some interparietal bone)] |
|  | archipterygial fin | absent |  |
|  | deltopectoral crest | present |  |
|  | dermal intracranial joint | present |  |
|  | entepicondyle of humerus | tapered |  |
|  | extrascapula | present |  |
|  | humerus diaphysis | cylindrical |  |
|  | individual digit of digitopodial skeleton | absent |  |
|  | median extrascapular | overlap with | lateral extrascapular |
|  | olecranon | absent |  |
|  | opercle | present |  |
|  | pectoral fin lepidotrichium | present |  |
|  | pelvic fin lepidotrichium | present |  |
|  | postaxial process of the ulnare | absent |  |
|  | proximal head of humerus | convex |  |
|  | radio-carpal joint | absent |  |
|  | radius bone | decreased length | humerus |
|  | radius bone | increased length | ulna |
|  | radius bone | blade-like |  |
|  | supinator process | present |  |
|  | supratemporal bone | separated from | interparietal bone |

**EQs from Garvey et al. (2005) for *Barameda***

| Entities | Qualities | Related Entities |
| --- | --- | --- |
| proximal head of humerus | separated from | ulnar condyle |
| neck of humerus | decreased length | ulnar condyle |
| proximal head of humerus | subcircular |  |
| neck of humerus | waisted |  |
| humerus | robust | ulnar condyle |
| [f521b0-e9c6-0131-44fd-005056010074 and (part_of some proximal head of humerus)] | conspicuous | ulnar condyle |
| [articular surface and (part_of some proximal head of humerus)] | sloped |  |
| radial head of humerus | aligned with |  |
| radial head of humerus | separated from |  |
| radial head of humerus | convex |  |
| ulnar condyle | convex |  |
| radial head of humerus | subcircular |  |
| ulnar condyle | subcircular | distal epiphysis of humerus |
| entepicondyle of humerus | obtuse angle to | humerus diaphysis |
| entepicondyle of humerus | increased size | pectoral fin distal radial bone 3 |
| entepicondyle of humerus | robust | intermedium (fore) |
| [dorsal margin and (part_of some entepicondyle of humerus)] | in contact with | ulna |
| dorsal ridge | in contact with | ulna |
| entepicondylar foramen | distal to | entepicondyle of humerus |
| [surface groove and (part_of some (distal region and (part_of some (humerus and (has_part some entepicondylar foramen)))))] | present | ulnare |
| [surface groove and (part_of some (distal region and (part_of some humerus)))] | decreased depth | [ulnar condyle and (bearer of some unfinished bone surface.)] |
| [surface groove and (part_of some (distal region and (part_of some humerus)))] | ventral to | [pectoral fin distal radial bone and (bearer of some d84a490-034c-0131-abbd-001ec9b0ea92)] |
| [surface groove and (part_of some (distal region and (part_of some humerus)))] | ventral to | [ulnar condyle and (bearer of some unfinished bone surface.)] |
| [entepicondylar foramen and (part_of some bone fossa)] | circular | ulnar condyle |
| bone fossa | ventral to | ulnar condyle |
| [muscle scar and (part_of some a914fa0-ea97-0131-411f-005056010073)] | increased depth |  |
| ectepicondylar flange | absent |  |
| ectepicondyle of humerus | present |  |
| ventral humeral ridge | in contact with |  |
| [ventral humeral ridge and (distal_to some entepicondyle of humerus)] | increased width |  |
| ventral humeral ridge | robust |  |
| [columnar area and (ventral_to some proximal head of humerus)] | present |  |
| [columnar area and (dorsal_to some dorsal ridge)] | present |  |
| [bone fossa and (part_of some columnar area)] | count |  |
| [bone fossa and (part_of some (columnar area and (UBERON_has_muscle_insertion some scapulohumeralis muscle)))] | present |  |
| neck of humerus | [shape and (not (elongated))] |  |
| neck of humerus | present |  |
| dorsal ridge | oriented towards |  |
| dorsal ridge | present |  |
| distal groove of humerus | present |  |
| [proximal surface and (part_of some proximal epiphysis of radius)] | articulated with |  |
| [proximal surface and (part_of some proximal epiphysis of radius)] | increased size |  |
| [distal surface and (part_of some proximal epiphysis of radius)] | concave |  |
| [distal surface and (part_of some proximal epiphysis of radius)] | elongated |  |
| [proximal surface and (part_of some proximal epiphysis of radius)] | circular |  |
| [proximal region and (part_of some (medial margin and (part_of some radius bone)))] | increased thickness |  |
| radius bone | [shape and (not (cylindrical))] |  |
| radius bone | increased length | ulnar condyle |
| radius bone | increased width | ulnar condyle |
| [distal surface and (part_of some ulna)] | concave | ulnare |
| ulna | robust | intermedium (fore) |
| ulna | square |  |
| [distal region and (part_of some ulna)] | increased width |  |
| [distal region and (part_of some ulna)] | articulated with |  |
| [distal region and (part_of some ulna)] | articulated with |  |
| [distal region and (part_of some ulna)] | splayed |  |
| [articular surface and (part_of some proximal epiphysis of ulna)] | increased size |  |
| [articular surface and (part_of some proximal epiphysis of ulna)] | concave |  |
| [articular surface and (part_of some distal epiphysis of ulna)] | flat |  |
| ulna | increased size |  |
| [distal region and (part_of some ulnare)] | broad |  |
| pisiform | absent |  |
| [distal region and (part_of some ulnare)] | articulated with |  |
| intermedium (fore) | increased length |  |
| intermedium (fore) | decreased width |  |
| intermedium (fore) | decreased width |  |
| intermedium (fore) | increased length |  |
| [distal region and (part_of some intermedium (fore))] | articulated with |  |
| [distal region and (part_of some intermedium (fore))] | splayed |  |
| intermedium (fore) | waisted |  |
| [dorsal surface and (part_of some intermedium (fore))] | ridged |  |
| pectoral fin distal radial bone | hourglass-shaped |  |
| pectoral fin lepidotrichium | overlapping |  |
| pectoral fin lepidotrichium | extends beyond |  |
| [pectoral fin lepidotrichium and (part_of some (distal margin and (part_of some fin)))] | hourglass-shaped |  |
| [transverse plane and (part_of some (proximal region and (part_of some pectoral fin lepidotrichium)))] | subcircular |  |
| [pectoral fin lepidotrichium and (part_of some (distal margin and (part_of some fin)))] | ridged |  |
| [proximal region and (part_of some pectoral fin lepidotrichium)] | convex |  |
| [distal region and (part_of some pectoral fin lepidotrichium)] | concave |  |
| pectoral fin lepidotrichium | tapered |  |
| pectoral fin lepidotrichium | branched |  |
| pectoral fin lepidotrichium | segmented |  |
| [pectoral fin lepidotrichium and (part_of some (proximal region and (part_of some fin)))] | branched |  |
| pectoral fin | increased length |  |
| pectoral fin | increased width | radial head of humerus |
| glenoid fossa | concave | ulnar condyle |
| pectoral fin | decreased flexibility | ulnar condyle |

**EQs from Matrices for *Panderichthys***

|  | Entities | Qualities | Related Entities |
| --- | --- | --- | --- |
| Clack et al. 2012 | interclavicle | decreased size | interclavicle |
|  | [articulation and (part_of some (anterior side and (part_of some anocleithrum)))] | present |  |
|  | [attachment site and (correlates_with some latissimus dorsi muscle)] | ridged |  |
|  | [proximal region and (part_of some ventral humeral ridge)] | present |  |
|  | anocleithrum | oblong |  |
|  | cleithrum | ornamented |  |
|  | coracoid foramen | present |  |
|  | entepicondyle of humerus | tapered |  |
|  | individual digit of digitopodial skeleton | absent |  |
|  | interclavicle | absent |  |
|  | interclavicle | concealed |  |
|  | intertemporal bone | present |  |
|  | latissimus dorsi process | absent |  |
|  | pectoral fin lepidotrichium | present |  |
|  | pelvic fin lepidotrichium | present |  |
|  | postbranchial lamina | present |  |
|  | posttemporal | present |  |
|  | prepectoral space | present |  |
|  | radius bone | increased length | ulna |
|  | scapular blade | absent |  |
|  | scapulocoracoid | increased size | scapulocoracoid |
|  | scapulocoracoid | platelike |  |
|  | supracleithrum | present |  |
|  | ventral humeral ridge | separated from | [anterior margin and (part_of some humerus)] |
|  | ventral humeral ridge | absent |  |
| Daeschler et al. 2006 | [anatomical margin and (part_of some (proximal region and (part_of some capitulum of humerus)))] | sharp |  |
|  | [anatomical projection and (part_of some (dorsal surface and (part_of some scapulocoracoid)))] | attached to | cleithrum |
|  | [anatomical projection and (part_of some (f9a6265-9f25-420d-b9d6-86d69315e0c0 and (part_of some ulnare)))] | absent |  |
|  | [anterior margin and (part_of some median extrascapular)] | increased length |  |
|  | [anterior margin and (part_of some ventral humeral ridge)] | adjacent to | proximal head of humerus |
|  | [articular surface and (part_of some (ulnare and (bearer of some multiple)))] | absent |  |
|  | [attachment quality and (part_of some cleithrum)] | concave |  |
|  | [muscle scar and (part_of some (proximal region and (part_of some capitulum of humerus)))] | present |  |
|  | [pectoral fin radial bone and (distal_to some ulnare)] | unbranched |  |
|  | [proximal region and (part_of some capitulum of humerus)] | increased size |  |
|  | [ventro-medial region and (part_of some coracoid plate)] | increased size |  |
|  | anal fin | absent |  |
|  | anocleithrum | ornamented |  |
|  | anocleithrum | exposed |  |
|  | archipterygial fin | absent |  |
|  | basal scute | absent |  |
|  | capitulum of humerus | distal orientation |  |
|  | cleithrum | ornamented |  |
|  | cleithrum | angle |  |
|  | coracoid plate | present |  |
|  | diphycercal tail | present |  |
|  | dorsal fin | absent |  |
|  | ectepicondylar flange | subterminal | epiphysis of humerus |
|  | humerus diaphysis | flattened |  |
|  | humerus diaphysis | rectangular |  |
|  | proximal head of humerus | elongated |  |
|  | proximal head of humerus | bifurcated |  |
|  | radius bone | decreased length | humerus |
|  | radius bone | blade-like |  |
|  | scapulocoracoid | size |  |
|  | scapulocoracoid | recurved |  |
|  | subscapular fossa | present |  |
|  | ulnar condyle | distal orientation |  |
|  | ventral humeral ridge | diagonal to | humerus |
|  | intercarpal joint | present |  |
|  | extrascapula | present |  |
|  | interclavicle | decreased size |  |
|  | median extrascapular | ventral to | lateral extrascapular |
|  | olecranon | absent |  |
|  | proximal head of humerus | strap-shaped |  |
|  | interclavicle | unornamented |  |
|  | glenoid fossa | lateral orientation |  |
|  | glenoid fossa | decreased elevation | clavicle |
| Ruta 2011 | [anatomical axis and (part_of some glenoid fossa)] | horizontal |  |
|  | [anatomical margin and (part_of some (clavicle and (continuous with some cleithrum)))] | concave |  |
|  | [anatomical projection and (part_of some (antero-ventral region and (part_of some cleithrum)))] | present |  |
|  | [anatomical projection and (part_of some (lateral region and (part_of some cleithrum)))] | present |  |
|  | [anatomical projection and (part_of some (posterior region and (part_of some cleithrum)))] | absent |  |
|  | [anatomical projection and (part_of some (posterior region and (part_of some tibia)))] | absent |  |
|  | [anterior margin and (part_of some cleithrum)] | increased angle to | horizontal plane |
|  | [anterior margin and (part_of some humerus)] | osseous |  |
|  | [anterior margin and (part_of some humerus)] | ridged |  |
|  | [articular surface and (part_of some distal epiphysis of tibia)] | absent |  |
|  | [basal region and (part_of some pectoral fin lepidotrichium)] | increased length |  |
|  | [articular surface and (part_of some proximal mesomere)] | present |  |
|  | [bone fossa and (part_of some ectepicondyle of humerus)] | absent |  |
|  | [bone fossa and (PHENOSCAPE_serves_as_attachment_site_for some muscle of shoulder)] | present |  |
|  | [clavicle and (has_part some (surface groove and (bearer of some anterolateral orientation)))] | absent |  |
|  | [clavicle and (in_right_side_of some multicellular organism)] | in contact with | [clavicle and (in_left_side_of some multicellular organism)] |
|  | [crest and (part_of some (humerus and (adjacent to some entepicondyle of humerus) and (adjacent to some ectepicondyle of humerus)))] | absent |  |
|  | [diaphysis and (part_of some cleithrum)] | present |  |
|  | [distal epiphysis of fibula and (bearer of some oblique orientation)] | absent |  |
|  | [distal margin and (part_of some ectepicondylar flange)] | aligned with | ulnar condyle |
|  | [distal region and (part_of some intermedium (fore))] | tapered |  |
|  | [dorsal region and (part_of some cleithrum)] | increased width |  |
|  | [distal region and (part_of some radius bone)] | tapered |  |
|  | [dorsal region and (part_of some cleithrum)] | spatulate |  |
|  | [ectepicondylar flange and (PHENOSCAPE_extends_to some distal epiphysis of humerus)] | present |  |
|  | [lateral surface and (part_of some fibula)] | convex |  |
|  | [muscle scar and (proximal_to some radial head of humerus)] | present |  |
|  | [ossification center and (part_of some scapula)] | undivided |  |
|  | [paired fin radial bone and (has_part some skeletal joint)] | absent |  |
|  | [posterior margin and (part_of some (entepicondyle of humerus and (bearer of some increased thickness)))] | absent |  |
|  | [posterior margin and (part_of some (ulna and (has_part some (anatomical projection and (bearer of some decreased size) and (bearer of some subcircular)))))] | absent |  |
|  | [posterior margin and (part_of some entepicondyle of humerus)] | size | [zone of bone organ and (part_of some (humerus diaphysis and (adjacent to some entepicondyle of humerus)))] |
|  | [posterior region and (part_of some interclavicle)] | increased length | [anterior region and (part_of some interclavicle)] |
|  | [postero-lateral margin and (part_of some entepicondyle of humerus)] | distal to | radial head of humerus |
|  | [postero-lateral margin and (part_of some entepicondyle of humerus)] | distal to | ulnar condyle |
|  | [scapulocoracoid and (has_part some (glenoid fossa and (bearer of some subterminal)))] | present |  |
|  | [scapulocoracoid and (has_part some attachment site)] | flush | [f9e4e6-ec85-434f-9708-93905386ad43 and (part_of some cleithrum)] |
|  | [sulcus and (part_of some (antero-dorsal region and (part_of some (flexor surface and (part_of some fibula)))))] | absent |  |
|  | [sulcus and (part_of some (humerus and (adjacent to some entepicondyle of humerus) and (adjacent to some ectepicondyle of humerus)))] | absent |  |
|  | [supraglenoid region and (bearer of some bone fossa)] | absent |  |
|  | [surface groove and (part_of some (ventral surface and (part_of some interclavicle)))] | absent |  |
|  | accessory foramina | present |  |
|  | accessory foramina | multiple |  |
|  | anocleithrum | present |  |
|  | anocleithrum | exposed |  |
|  | ascending process of clavicle | absent |  |
|  | basal scute | absent |  |
|  | clavicle | flush | cleithrum |
|  | clavicle | rectangular |  |
|  | cleithrum | attached to | scapulocoracoid |
|  | cleithrum | [shape and (not (waisted))] |  |
|  | condyle of femur | width |  |
|  | coracoid plate | increased size |  |
|  | deltoid process | associated with | dorsal ridge |
|  | deltoid process | present |  |
|  | deltoid process | decreased thickness |  |
|  | cleithrum | ornamented |  |
|  | diaphysis of tibia | decreased width | diaphysis of fibula |
|  | distal epiphysis of humerus | aligned with | proximal epiphysis of humerus |
|  | dorsal iliac process | absent |  |
|  | ectepicondylar flange | present |  |
|  | ectepicondylar foramen | present |  |
|  | entepicondylar foramen | elliptic |  |
|  | entepicondylar foramen | present |  |
|  | entepicondylar foramen | subcircular |  |
|  | entepicondyle of humerus | present |  |
|  | entepicondyle of humerus | blunt |  |
|  | fibula | [shape and (not (waisted))] |  |
|  | fibula | [width and (PATO_increased_in_magnitude_relative_to some (length and (inheres_in some fibula)))] |  |
|  | entepicondyle of humerus | subcircular |  |
|  | glenoid fossa | lateral orientation |  |
|  | head of femur | decreased size |  |
|  | glenoid fossa | ventral to | clavicle |
|  | hindlimb interepipodial space | absent |  |
|  | humerus | [length and (PATO_decreased_in_magnitude_relative_to some (width and (inheres_in some humerus)))] |  |
|  | humerus | dorso-ventrally flattened |  |
|  | humerus diaphysis | [shape and (not (waisted))] |  |
|  | interclavicle | unornamented |  |
|  | interclavicle | decreased size |  |
|  | humerus | increased length | trunk vertebra |
|  | interclavicle | scute-like |  |
|  | interclavicle | [width and (PATO_increased_in_magnitude_relative_to some (length and (inheres_in some interclavicle)))] |  |
|  | interclavicle | rhomboid |  |
|  | intermedium (fore) | spindle-shaped |  |
|  | intermedium (fore) | present |  |
|  | latissimus dorsi process | absent |  |
|  | manual digit digitopodial skeleton | absent |  |
|  | metacarpal bone | absent |  |
|  | metatarsal bone | absent |  |
|  | neck of humerus | absent |  |
|  | olecranon | absent |  |
|  | paired fin radial bone | present |  |
|  | parasternal process | absent |  |
|  | pectoral fin radial bone | unbranched |  |
|  | pectoral process of humerus | present |  |
|  | pectoral process of humerus | triangular |  |
|  | pectoral process of humerus | decreased thickness |  |
|  | pedal digit digitopodial skeleton | absent |  |
|  | postaxial process of the fibula | absent |  |
|  | postaxial process of the ulnare | absent |  |
|  | postbranchial lamina | present |  |
|  | process 2 | absent |  |
|  | process 3 | absent |  |
|  | process 4 | absent |  |
|  | proximal epiphysis of radius | decreased size |  |
|  | proximal head of humerus | strap-shaped |  |
|  | proximal epiphysis of tibia | increased width | distal epiphysis of tibia |
|  | proximal head of humerus | bifurcated |  |
|  | proximal head of humerus | elongated |  |
|  | radial head of humerus | terminal |  |
|  | radius bone | increased length | ulna |
|  | radius bone | [length and (PATO_increased_in_magnitude_relative_to some (width and (inheres_in some (d573b0-034c-0131-abbd-001ec9b0ea92 and (part_of some radius bone)))))] |  |
|  | radius bone | present |  |
|  | scapular blade | present |  |
|  | scapulocoracoid | concealed by | cleithrum |
|  | scapular blade | decreased size |  |
|  | supracleithrum | decreased size |  |
|  | subscapular fossa | present |  |
|  | supinator process | absent |  |
|  | supracleithrum | present |  |
|  | supraglenoid foramen | size |  |
|  | supraglenoid foramen | present |  |
|  | supraglenoid foramen | subcircular |  |
|  | tibia | [shape and (not (waisted))] |  |
|  | tibia | [width and (PATO_decreased_in_magnitude_relative_to some (length and (inheres_in some tibia)))] |  |
|  | ulna | [width and (PATO_similar_in_magnitude_relative_to some (length and (inheres_in some ulna)))] |  |
|  | tibia | decreased length | femur |
|  | ulnar facet of the humerus | undivided | radial facet |
|  | ventral humeral ridge | extends to | [postero-medial margin and (part_of some entepicondyle of humerus)] |
|  | ventral humeral ridge | oblique orientation |  |
|  | ventral humeral ridge | present |  |
| Swartz 2012 | [anatomical margin and (part_of some cleithrum)] | concave |  |
|  | [anatomical projection and (part_of some (posterior margin and (part_of some body of rib)))] | decreased width |  |
|  | [anatomical region and (adjacent to some (facet and (part_of some radius bone)))] | increased size |  |
|  | [anterior margin and (part_of some median extrascapular)] | increased length |  |
|  | [anatomical region and (adjacent to some (facet and (part_of some radius bone)))] | sharp |  |
|  | [anterior margin and (part_of some ventral humeral ridge)] | adjacent to | proximal epiphysis of humerus |
|  | [basal region and (part_of some paired fin lepidotrichium)] | [shape and (not (elongated))] |  |
|  | [articulation and (part_of some (anterior side and (part_of some anocleithrum)))] | present |  |
|  | [extratemporal bone and (posterior_to some spiracle)] | absent |  |
|  | [facet and (part_of some radius bone)] | distal orientation |  |
|  | [facet and (part_of some ulna)] | distal orientation |  |
|  | [muscle scar and (part_of some (facet and (part_of some radius bone)))] | present |  |
|  | [pectoral fin radial bone and (continuous with some ulnare)] | absent |  |
|  | [pectoral fin radial bone and (distal_to some ulnare)] | unbranched |  |
|  | [posterior margin and (part_of some tabular bone)] | level with | [posterior margin and (part_of some interparietal bone)] |
|  | anal fin | absent |  |
|  | anocleithrum | exposed |  |
|  | anocleithrum | oblong |  |
|  | archipterygial fin | absent |  |
|  | basicranial fenestra | present |  |
|  | body of rib | increased length | [bony vertebral centrum and (has_part some diameter)] |
|  | body of rib | straight |  |
|  | cleithrum | ornamented |  |
|  | cleithrum | [orientation and (not (vertical))] |  |
|  | coracoid plate | increased size |  |
|  | coracoid foramen | present |  |
|  | deltopectoral crest | present |  |
|  | dermal intracranial joint | absent |  |
|  | diphycercal tail | present |  |
|  | dorsal fin | absent |  |
|  | ectepicondylar flange | proximal to | condyle of humerus |
|  | entepicondyle of humerus | tapered |  |
|  | extrascapula | present |  |
|  | glenoid fossa | parallel to | clavicle |
|  | glenoid fossa | lateral orientation |  |
|  | glenoid fossa | [height and (PATO_decreased_in_magnitude_relative_to some (width and (inheres_in some glenoid fossa)))] |  |
|  | humerus diaphysis | flattened |  |
|  | humerus diaphysis | rectangular |  |
|  | individual digit of digitopodial skeleton | absent |  |
|  | interclavicle | decreased size |  |
|  | interclavicle | unornamented |  |
|  | interclavicle | absent |  |
|  | interclavicle | concealed |  |
|  | median extrascapular | overlap with | lateral extrascapular |
|  | olecranon | absent |  |
|  | opercle | present |  |
|  | pectoral fin lepidotrichium | present |  |
|  | pelvic fin lepidotrichium | present |  |
|  | postaxial process of the fibula | decreased size |  |
|  | postaxial process of the fibula | present |  |
|  | postaxial process of the ulnare | absent |  |
|  | postbranchial lamina | present |  |
|  | posttemporal | present |  |
|  | preopercle | present |  |
|  | proximal head of humerus | convex |  |
|  | radio-carpal joint | absent |  |
|  | radius bone | increased length | ulna |
|  | radius bone | increased length | humerus |
|  | radius bone | blade-like |  |
|  | rib | [structure and (not (overlapping))] |  |
|  | rib | decreased size |  |
|  | scapular blade | absent |  |
|  | scapulocoracoid | increased size |  |
|  | scapulocoracoid | platelike |  |
|  | spiracular notch | decreased width |  |
|  | supinator process | present |  |
|  | supracleithrum | present |  |
|  | supraneural bone | absent |  |
|  | supratemporal bone | separated from | interparietal bone |
| Vallin and Laurin 2004 | [dorsal region and (part_of some cleithrum)] | increased size |  |
|  | anocleithrum | present |  |
|  | anterior humeral ridge | present |  |
|  | clavicle | present |  |
|  | ectepicondyle of humerus | decreased height |  |
|  | humerus | present |  |
|  | lateral extrascapular | present |  |
|  | manual digit digitopodial skeleton | absent |  |
|  | median extrascapular | present |  |
|  | olecranon | absent |  |
|  | parasternal process | absent |  |
|  | pedal digit digitopodial skeleton | absent |  |
|  | postbranchial lamina | present |  |
|  | radius bone | separated from | ulna |
|  | scapulocoracoid | fused with | cleithrum |
|  | supinator process | decreased size |  |
|  | talus | absent |  |
|  | ulna | decreased length | radius bone |

**EQs from Boisvert (2005) for *Panderichthys***

| Entities | Qualities | Related Entities |
| --- | --- | --- |
| pelvic girdle skeleton | decreased size |  |
| [anatomical surface and (part_of some (anterior region and (part_of some pelvic girdle skeleton)))] | increased thickness |  |
| pelvic girdle skeleton | flat |  |
| pelvic girdle skeleton | clavate |  |
| [anatomical surface and (part_of some (anterior region and (part_of some pelvic girdle skeleton)))] | unfinished bone surface. |  |
| [anatomical surface and (part_of some (posterior region and (part_of some pelvic girdle skeleton)))] | finished bone surface |  |
| pelvic fin | articulated with | [posterior margin and (part_of some pelvic girdle skeleton)] |
| iliac ramus | absent |  |
| femur | overlapping | fibula |
| femur | flat |  |
| postaxial process of the femur | decreased width |  |
| [proximal region and (part_of some femur)] | decreased width |  |
| [anatomical projection and (part_of some femur)] | extends to | distal epiphysis of femur |
| [anatomical projection and (part_of some femur)] | anterior to | [epipodial facet and (part_of some femur)] |
| fibula | decreased length | tibia |
| fibula | increased width | tibia |
| fibula | platelike |  |
| [proximal region and (part_of some fibula)] | overlapped by | dermal scale |
| tibia | increased length |  |
| tibia | decreased width |  |
| tibia | cylindrical |  |
| fibulare | overlapped by | lepidotrichium |
| hindlimb intermedium | overlapped by | lepidotrichium |
| pelvic fin skeleton | extends beyond | [proximal region and (part_of some lepidotrichium)] |
| ilium | absent |  |
| scapulocoracoid | size |  |
| acetabular fossa | posterior orientation |  |
| glenoid fossa | posterolateral orientation |  |
| ulna | parallel to | radius bone |
| fibula | parallel to | tibia |
| ulna | flattened |  |
| fibula | flattened |  |
| postaxial process of the ulnare | absent |  |
| postaxial process of the fibula | absent |  |
| tibia | flattened |  |
| humerus | flattened |  |
| adductor blade | absent |  |
| adductor crest | absent |  |
| fibula | increased width | tibia |
| fibulare | increased width | hindlimb intermedium |
| ulnare | increased width | intermedium (fore) |
| elbow joint | decreased mobility |  |
| knee joint | decreased mobility |  |
| [articular surface and (part_of some hindlimb skeleton)] | broad |  |
| [articular surface and (part_of some forelimb skeleton)] | broad |  |
| pectoral fin | increased size | pelvic fin |

**EQs from Boisvert et al. (2008) for *Panderichthys***

| Entities | Qualities | Related Entities |
| --- | --- | --- |
| pectoral fin | overlapped by | dermal scale |
| pectoral fin | overlapped by | lepidotrichium |
| pectoral fin | aligned with | main body axis |
| [transverse plane and (part_of some (dorsal side and (part_of some (distal region and (part_of some pectoral fin)))))] | convex |  |
| [transverse plane and (part_of some (ventral side and (part_of some (distal region and (part_of some pectoral fin)))))] | concave |  |
| anocleithrum | increased length |  |
| [proximal region and (part_of some anocleithrum)] | overlapped by | supracleithrum |
| ectepicondyle of humerus | parallel to | [f517ba0-034c-0131-abbd-001ec9b0ea92 and (part_of some humerus)] |
| ectepicondyle of humerus | linear |  |
| ectepicondyle of humerus | decreased height |  |
| ectepicondyle of humerus | anterior to | radio-ulnar joint |
| ectepicondyle of humerus | decreased length |  |
| ventral humeral ridge | extends to | [proximal region and (part_of some entepicondyle of humerus)] |
| ventral humeral ridge | increased height |  |
| ventral humeral ridge | oblique orientation |  |
| [ventral side and (part_of some radius bone)] | convex |  |
| radius bone | slender |  |
| radius bone | falciform |  |
| [lateral margin and (part_of some radius bone)] | increased thickness |  |
| [ventral surface and (part_of some radius bone)] | smooth |  |
| [dorsal surface and (part_of some radius bone)] | smooth |  |
| [proximal region and (part_of some ulnare)] | articulated with | ulna |
| [distal region and (part_of some ulnare)] | articulated with | radial bone |
| ulnare | decreased size |  |
| ulnare | flattened |  |
| [articulation and (part_of some intermedium (fore))] | aligned with | [anatomical projection and (UBERON_in_lateral_side_of some ulna)] |
| intermedium (fore) | cylindrical |  |
| intermedium (fore) | flattened |  |
| intermedium (fore) | bifurcated |  |
| pectoral fin distal radial bone | count |  |
| ulnare | separated from | pectoral fin distal radial bone |
| pectoral fin distal radial bone 1 | separated from | pectoral fin distal radial bone 2 |
| pectoral fin distal radial bone | dorso-ventrally flattened |  |
| [pectoral fin distal radial bone and (bearer of some width)] | variant |  |
| ulna | increased size | ulnare |
| ulna | increased length | ulnare |
| distal carpal endochondral element | [position and (not (distal to))] | ulnare |
| pectoral fin distal radial bone | fan-shaped |  |
| pectoral fin distal radial bone | aligned with | ulnare |
| [pectoral fin distal radial bone and (adjacent to some a2b6060-034c-0131-abbd-001ec9b0ea92)] | Count |  |
| [pectoral fin distal radial bone and (adjacent to some f517ba0-034c-0131-abbd-001ec9b0ea92)] | Count |  |
| [pectoral fin distal radial bone and (adjacent to some a2b6060-034c-0131-abbd-001ec9b0ea92)] | [structure and (not (articulated with))] | [bone element and (part_of some metapterygial axis)] |

**EQs from Boisvert (2009) for *Panderichthys***

| Entities | Qualities | Related Entities |
| --- | --- | --- |
| [dorsal surface and (part_of some proximal epiphysis of humerus)] | anterior orientation |  |
| ulnar facet of the humerus | ventral orientation |  |
| [radial facet and (part_of some distal epiphysis of humerus)] | Present |  |
| [ulnar facet of the humerus and (part_of some distal epiphysis of humerus)] | Present |  |
| radial facet | ventral orientation |  |
| ulnar facet of the humerus | separated from | radial facet |
| radial facet | obtuse angle to |  |
| ulnar facet of the humerus | parallel to | radius bone |
| entepicondyle of humerus | obtuse angle to | humerus diaphysis |
| humerus | L-shaped |  |
| entepicondyle of humerus | decreased thickness |  |
| entepicondyle of humerus | curved ventral |  |
| entepicondyle of humerus | Perforate |  |
| entepicondyle of humerus | blade-like |  |
| [a2b6060-034c-0131-abbd-001ec9b0ea92 and (in_anterior_side_of some humerus)] | curved ventral |  |
| deltoid process | in contact with | supinator process |
| supinator process | Present |  |
| deltoid process | Present |  |
| supinator process | acute angle to | deltoid process |
| ectepicondyle of humerus | increased width |  |
| ectepicondyle of humerus | Flat |  |
| ectepicondyle of humerus | [position and (not (extends to))] | distal epiphysis of humerus |
| ectepicondylar foramen | diagonal to | [proximal region and (part_of some ectepicondyle of humerus)] |
| [excurrent foramen of ectepicondylar foramen and (part_of some ectepicondylar foramen)] | adjacent to | supinator process |
| [incurrent foramen of ectepicondylar formen and (part_of some ectepicondylar foramen)] | adjacent to | ectepicondyle of humerus |
| [excurrent foramen of ectepicondylar foramen and (part_of some ectepicondylar foramen)] | adjacent to | ectepicondyle of humerus |
| [incurrent foramen of ectepicondylar formen and (part_of some ectepicondylar foramen)] | adjacent to | [post-axial region and (part_of some distal epiphysis of humerus)] |
| ventral humeral ridge | extends to | ectepicondyle of humerus |
| ventral humeral ridge | increased height |  |
| ventral humeral ridge | decreased width |  |
| ventral humeral ridge | Centered |  |
| ventral humeral ridge | parallel to | [post-axial region and (part_of some humerus)] |
| pectoral process of humerus | Absent |  |
| [incurrent foramen of ectepicondylar formen and (part_of some entepicondyle fossa)] | Present |  |
| [surface groove and (part_of some (post-axial region and (part_of some distal epiphysis of humerus)))] | Present |  |
| [excurrent foramen of ectepicondylar foramen and (part_of some entepicondyle fossa)] | anterior to | ulnar condyle |
| radial facet | separated from | ulnar facet of the humerus |
| ulnar facet of the humerus | aligned with | ectepicondyle of humerus |
| humerus | L-shaped |  |
| entepicondyle of humerus | decreased angle to | [post-axial region and (part_of some humerus diaphysis)] |
| humerus | [length and (PATO_increased_in_magnitude_relative_to some (width and (inheres_in some humerus)))] |  |
| latissimus dorsi process | Present |  |
| latissimus dorsi process | associated with | humerus diaphysis |
| latissimus dorsi process | increased height |  |
| [muscle scar and (part_of some scapulohumeralis muscle)] | Present |  |
| deltoid process | separated from | supinator process |
| ectepicondyle of humerus | separated from | supinator process |
| ectepicondyle of humerus | separated from | deltoid process |
| pectoral process of humerus | Absent |  |
| ventral humeral ridge | transverse orientation |  |
| ventral humeral ridge | increased height |  |
| ventral humeral ridge | Perforate |  |
| [ventral humeral ridge and (UBERON_distally_connected_to some entepicondyle of humerus)] | Subterminal |  |
| ventral humeral ridge | Present |  |
| entepicondyle of humerus | decreased thickness |  |
| entepicondyle of humerus | blade-like |  |
| entepicondyle of humerus | ventral orientation |  |
| humerus | dorso-ventrally flattened |  |

**EQs from Matrices for *Tiktaalik***

| Matrix | Entities | Qualities | Related Entities |
| --- | --- | --- | --- |
| Clack et al. 2012 | [articulation and (part_of some (anterior side and (part_of some anocleithrum)))] | Present |  |
|  | anocleithrum | Oblong |  |
|  | cleithrum | ornamented |  |
|  | postbranchial lamina | Present |  |
|  | individual digit of digitopodial skeleton | Absent |  |
|  | entepicondyle of humerus | Tapered |  |
|  | interclavicle | Absent |  |
|  | interclavicle | decreased size |  |
|  | interclavicle | concealed |  |
|  | pectoral fin lepidotrichium | Present |  |
|  | pelvic fin lepidotrichium | Present |  |
|  | radius bone | increased length |  |
|  | scapular blade | Absent |  |
|  | coracoid foramen | Present |  |
|  | scapulocoracoid | increased size |  |
|  | scapulocoracoid | platelike |  |
|  | subscapular fossa | Broad |  |
|  | subscapular fossa | decreased depth |  |
|  | ventral humeral ridge | Absent |  |
|  | [proximal region and (part_of some ventral humeral ridge)] | Present |  |
|  | prepectoral space | Present |  |
|  | ventral humeral ridge | separated from |  |
|  | [attachment site and (correlates_with some latissimus dorsi muscle)] | Ridged |  |
|  | latissimus dorsi process | Absent |  |
|  | [anatomical conduit and (part_of some ventral humeral ridge)] | Multiple |  |
| Daeschler et al. 2006 | [attachment quality and (part_of some cleithrum)] | Concave |  |
|  | anocleithrum | exposed |  |
|  | archipterygial fin | Absent |  |
|  | humerus diaphysis | flattened |  |
|  | humerus diaphysis | rectangular | epiphysis of humerus |
|  | anal fin | Absent | cleithrum |
|  | dorsal fin | Absent | proximal head of humerus |
|  | basal scute | Absent |  |
|  | extrascapula | Absent |  |
|  | scapulocoracoid | Size | clavicle |
|  | scapulocoracoid | recurved |  |
|  | intercarpal joint | Present | humerus |
|  | [articular surface and (part_of some (ulnare and (bearer of some multiple)))] | Present |  |
|  | [anatomical projection and (part_of some (f9a6265-9f25-420d-b9d6-86d69315e0c0 and (part_of some ulnare)))] | Absent |  |
|  | [pectoral fin radial bone and (distal_to some ulnare)] | branched |  |
|  | radius bone | blade-like |  |
|  | olecranon | Absent |  |
|  | [anterior margin and (part_of some ventral humeral ridge)] | adjacent to |  |
|  | ventral humeral ridge | diagonal to |  |
|  | capitulum of humerus | ventral orientation |  |
|  | ulnar condyle | distal orientation |  |
|  | ectepicondylar flange | distal to |  |
|  | radius bone | decreased length |  |
|  | [anatomical margin and (part_of some (proximal region and (part_of some capitulum of humerus)))] | Sharp |  |
|  | [muscle scar and (part_of some (proximal region and (part_of some capitulum of humerus)))] | Present |  |
|  | [proximal region and (part_of some capitulum of humerus)] | increased size |  |
|  | [ventro-medial region and (part_of some coracoid plate)] | increased size |  |
|  | coracoid plate | Present |  |
|  | glenoid fossa | decreased elevation |  |
|  | cleithrum | Angle |  |
|  | cleithrum | ornamented |  |
|  | anocleithrum | ornamented |  |
|  | [anatomical projection and (part_of some (dorsal surface and (part_of some scapulocoracoid)))] | attached to | humerus |
|  | subscapular fossa | Present |  |
|  | glenoid fossa | lateral orientation |  |
|  | proximal head of humerus | strap-shaped |  |
|  | proximal head of humerus | elongated |  |
|  | proximal head of humerus | bifurcated |  |
| Ruta 2011 | postbranchial lamina | Present |  |
|  | [scapulocoracoid and (has_part some attachment site)] | Flush | [f9e4e6-ec85-434f-9708-93905386ad43 and (part_of some cleithrum)] |
|  | cleithrum | attached to | scapulocoracoid |
|  | [anterior margin and (part_of some cleithrum)] | increased angle to | horizontal plane |
|  | cleithrum | ornamented |  |
|  | [dorsal region and (part_of some cleithrum)] | increased width |  |
|  | [dorsal region and (part_of some cleithrum)] | spatulate |  |
|  | [anatomical margin and (part_of some (clavicle and (continuous with some cleithrum)))] | Concave |  |
|  | cleithrum | [shape and (not (waisted))] |  |
|  | [anatomical projection and (part_of some (posterior region and (part_of some cleithrum)))] | Absent |  |
|  | [anatomical projection and (part_of some (antero-ventral region and (part_of some cleithrum)))] | present |  |
|  | [anatomical projection and (part_of some (lateral region and (part_of some cleithrum)))] | Present |  |
|  | [diaphysis and (part_of some cleithrum)] | Present |  |
|  | [clavicle and (has_part some (surface groove and (bearer of some anterolateral orientation)))] | Absent |  |
|  | clavicle | equilateral triangular |  |
|  | clavicle | rectangular |  |
|  | ascending process of clavicle | Absent |  |
|  | clavicle | Flush | cleithrum |
|  | supraglenoid foramen | Size |  |
|  | supraglenoid foramen | Present |  |
|  | supraglenoid foramen | subcircular |  |
|  | infraglenoid buttress | Present |  |
|  | glenoid fossa | lateral orientation |  |
|  | subscapular fossa | Present |  |
|  | scapular blade | Present |  |
|  | scapular blade | decreased size |  |
|  | glenoid fossa | ventral to | clavicle |
|  | scapulocoracoid | concealed by | cleithrum |
|  | coracoid plate | increased size |  |
|  | [supraglenoid region and (bearer of some bone fossa)] | Absent |  |
|  | [anatomical axis and (part_of some glenoid fossa)] | horizontal |  |
|  | anocleithrum | Present |  |
|  | anocleithrum | exposed |  |
|  | supracleithrum | decreased size |  |
|  | supracleithrum | Present |  |
|  | ventral humeral ridge | Present |  |
|  | ventral humeral ridge | oblique orientation |  |
|  | latissimus dorsi process | Absent |  |
|  | entepicondylar foramen | Elliptic |  |
|  | entepicondylar foramen | Present |  |
|  | entepicondylar foramen | subcircular |  |
|  | [ectepicondylar flange and (PHENOSCAPE_extends_to some distal epiphysis of humerus)] | Present |  |
|  | radial head of humerus | terminal |  |
|  | radial head of humerus | ventral orientation |  |
|  | humerus | [length and (PATO_decreased_in_magnitude_relative_to some (width and (inheres_in some humerus)))] |  |
|  | [postero-lateral margin and (part_of some (entepicondyle of humerus and (bearer of some concave)))] | Present |  |
|  | accessory foramina | Present |  |
|  | accessory foramina | Multiple |  |
|  | process 2 | Absent |  |
|  | humerus | dorso-ventrally flattened |  |
|  | [bone fossa and (PHENOSCAPE_serves_as_attachment_site_for some muscle of shoulder)] | Present |  |
|  | [muscle scar and (proximal_to some radial head of humerus)] | Present |  |
|  | entepicondyle of humerus | Blunt |  |
|  | entepicondyle of humerus | subcircular |  |
|  | proximal head of humerus | bifurcated |  |
|  | proximal head of humerus | elongated |  |
|  | proximal head of humerus | strap-shaped |  |
|  | ulnar facet of the humerus | separated from | radial facet |
|  | [anatomical projection and (part_of some (ventral region and (part_of some entepicondyle of humerus)))] | Present |  |
|  | [anterior margin and (part_of some humerus)] | Osseous |  |
|  | [anterior margin and (part_of some humerus)] | Ridged |  |
|  | distal epiphysis of humerus | aligned with | proximal epiphysis of humerus |
|  | [posterior margin and (part_of some entepicondyle of humerus)] | Size | [zone of bone organ and (part_of some (humerus diaphysis and (adjacent to some entepicondyle of humerus)))] |
|  | neck of humerus | Absent |  |
|  | [crest and (part_of some (humerus and (adjacent to some entepicondyle of humerus) and (adjacent to some ectepicondyle of humerus)))] | Absent |  |
|  | [sulcus and (part_of some (humerus and (adjacent to some entepicondyle of humerus) and (adjacent to some ectepicondyle of humerus)))] | Absent |  |
|  | [posterior margin and (part_of some (entepicondyle of humerus and (bearer of some increased thickness)))] | Absent |  |
|  | ventral humeral ridge | extends to | [postero-medial margin and (part_of some entepicondyle of humerus)] |
|  | entepicondyle of humerus | Present |  |
|  | [articular surface and (part_of some proximal mesomere)] | Present |  |
|  | process 3 | Absent |  |
|  | process 4 | Absent |  |
|  | pectoral process of humerus | Present |  |
|  | pectoral process of humerus | triangular |  |
|  | pectoral process of humerus | decreased thickness |  |
|  | deltoid process | associated with | dorsal ridge |
|  | deltoid process | Present |  |
|  | deltoid process | decreased thickness |  |
|  | [bone fossa and (part_of some ectepicondyle of humerus)] | Absent |  |
|  | radius bone | increased length | ulna |
|  | [distal region and (part_of some radius bone)] | truncated |  |
|  | radius bone | [length and (PATO_increased_in_magnitude_relative_to some (width and (inheres_in some (d573b0-034c-0131-abbd-001ec9b0ea92 and (part_of some radius bone)))))] |  |
|  | radius bone | Present |  |
|  | proximal epiphysis of radius | decreased size |  |
|  | olecranon | Absent |  |
|  | ulna | [width and (PATO_similar_in_magnitude_relative_to some (length and (inheres_in some ulna)))] |  |
|  | [posterior margin and (part_of some (ulna and (has_part some (anatomical projection and (bearer of some decreased size) and (bearer of some subcircular)))))] | Absent |  |
|  | postaxial process of the ulnare | Absent |  |
|  | intermedium (fore) | Present |  |
|  | intermedium (fore) | cylindrical |  |
|  | manual digit digitopodial skeleton | Absent |  |
|  | metacarpal bone | Absent |  |
|  | basal scute | Absent |  |
|  | [paired fin radial bone and (has_part some skeletal joint)] | Present |  |
|  | paired fin radial bone | Present |  |
|  | pectoral fin radial bone | bifurcated |  |
|  | [basal region and (part_of some pectoral fin lepidotrichium)] | increased length |  |
|  | mesomere 4 | decreased length |  |
|  | mesomere 4 | Broad |  |
|  | [posterior region and (part_of some mesomere 5)] | Broad |  |
| Swartz 2012 | basicranial fenestra | Present |  |
|  | dermal intracranial joint | Absent |  |
|  | supratemporal bone | separated from | interparietal bone |
|  | [posterior margin and (part_of some tabular bone)] | level with | [posterior margin and (part_of some interparietal bone)] |
|  | [extratemporal bone and (posterior_to some spiracle)] | Absent |  |
|  | preopercle | increased size |  |
|  | preopercle | Present |  |
|  | opercle | Absent |  |
|  | spiracular notch | increased width |  |
|  | extrascapula | Absent |  |
|  | posttemporal | Absent |  |
|  | supracleithrum | Present |  |
|  | anocleithrum | exposed |  |
|  | [articulation and (part_of some (anterior side and (part_of some anocleithrum)))] | Present |  |
|  | anocleithrum | Oblong |  |
|  | cleithrum | [orientation and (not (vertical))] |  |
|  | postbranchial lamina | Present |  |
|  | [anatomical margin and (part_of some cleithrum)] | Concave |  |
|  | coracoid foramen | Present |  |
|  | scapulocoracoid | increased size |  |
|  | scapulocoracoid | platelike |  |
|  | coracoid plate | increased size |  |
|  | scapular blade | Absent |  |
|  | proximal head of humerus | Convex |  |
|  | glenoid fossa | parallel to | clavicle |
|  | glenoid fossa | lateral orientation |  |
|  | interclavicle | Absent |  |
|  | interclavicle | decreased size |  |
|  | interclavicle | unornamented |  |
|  | interclavicle | concealed |  |
|  | archipterygial fin | Absent |  |
|  | entepicondyle of humerus | Tapered |  |
|  | humerus diaphysis | flattened |  |
|  | humerus diaphysis | rectangular |  |
|  | deltopectoral crest | Present |  |
|  | supinator process | Present |  |
|  | [anterior margin and (part_of some ventral humeral ridge)] | adjacent to | proximal epiphysis of humerus |
|  | ectepicondylar flange | distal to | condyle of humerus |
|  | radius bone | increased length | ulna |
|  | [facet and (part_of some radius bone)] | ventral orientation |  |
|  | [anatomical region and (adjacent to some (facet and (part_of some radius bone)))] | increased size |  |
|  | [anatomical region and (adjacent to some (facet and (part_of some radius bone)))] | Sharp |  |
|  | [muscle scar and (part_of some (facet and (part_of some radius bone)))] | Present |  |
|  | radius bone | blade-like |  |
|  | radius bone | decreased length | humerus |
|  | [facet and (part_of some ulna)] | distal orientation |  |
|  | olecranon | Absent |  |
|  | radio-carpal joint | Present |  |
|  | [pectoral fin radial bone and (continuous with some ulnare)] | Present |  |
|  | postaxial process of the ulnare | Absent |  |
|  | [pectoral fin radial bone and (distal_to some ulnare)] | branched |  |
|  | individual digit of digitopodial skeleton | Absent |  |
|  | pectoral fin lepidotrichium | Present |  |
|  | pelvic fin lepidotrichium | Present |  |
|  | [basal region and (part_of some paired fin lepidotrichium)] | elongated |  |
|  | rib | increased size |  |
|  | rib | overlapping |  |
|  | body of rib | Straight |  |
|  | [anatomical projection and (part_of some (posterior margin and (part_of some body of rib)))] | decreased width |  |
|  | [anatomical conduit and (part_of some iliac neck)] | Absent |  |
|  | anal fin | Absent |  |
|  | dorsal fin | Absent |  |
|  | cleithrum | ornamented |  |

**EQs from Shubin et al. (2006) for *Tiktaalik***

| Entities | Qualities | Related Entities |
| --- | --- | --- |
| coracoid bone | ventral to | multicellular organism |
| ectepicondyle of humerus | extends beyond | entepicondyle of humerus |
| pectoral girdle skeleton | separated from | skull |
| metapterygial axis | extends beyond | ulnare |
| pectoral fin lepidotrichium | surrounding | forelimb endochondral element |
| ectepicondyle of humerus | extends beyond | radial head of humerus |
| radial facet | separated from | ulnar facet of the humerus |
| radial facet | anteroventral to | ulnar facet of the humerus |
| radial facet | decreased size | ulnar facet of the humerus |
| scapular process | flush | [medial marginal vein and (part_of some cleithrum)] |
| [musculotendinous bundle and (UBERON_has_muscle_origin some (dorso-medial region and (part_of some coracoid plate)))] | extends to | [musculotendinous bundle and (UBERON_has_muscle_insertion some (ventral surface and (part_of some humerus)))] |
| ectepicondyle of humerus | increased size |  |
| ectepicondyle of humerus | distal orientation |  |
| ventral humeral ridge | diagonal to | [proximal-distal axis and (part_of some humerus)] |
| ventral humeral ridge | extends to | [distal margin and (part_of some entepicondyle of humerus)] |
| [posterior region and (part_of some radial facet)] | distal orientation |  |
| glenoid fossa | convex |  |
| [facet and (part_of some proximal epiphysis of humerus)] | concave |  |
| opercle | absent |  |
| subopercle | absent |  |
| extrascapula | absent |  |
| coracoid foramen | increased size |  |
| coracoid bone | increased thickness |  |
| ventral humeral ridge | robust |  |
| scapular process | increased width |  |
| coracoid bone | horizontal |  |
| coracoid bone | platelike |  |
| postaxial process of the ulnare | absent |  |
| [articular surface and (part_of some ulnare)] | multiple |  |
| [articulation and (part_of some (distal region and (part_of some forelimb endochondral element)))] | increased mobility |  |
| olecranon | absent |  |
| [articular surface and (in_distal_side_of some ulnare)] | extends to | [dorsal surface and (part_of some ulnare)] |
| [articular surface and (in_distal_side_of some pectoral fin proximal radial bone)] | extends to | [dorsal surface and (part_of some pectoral fin proximal radial bone)] |
| [pectoral fin lepidotrichium and (UBERON_attaches_to some (forelimb endochondral element and (in_anterior_side_of some forelimb)))] | increased thickness | [pectoral fin lepidotrichium and (UBERON_attaches_to some (forelimb endochondral element and (in_posterior_side_of some forelimb)))] |
| [articular surface and (in_distal_side_of some intermedium (fore))] | extends to | [dorsal surface and (part_of some intermedium (fore))] |
| metapterygial axis | centered |  |
| coracoid foramen | increased size |  |
| [articular surface and (part_of some pectoral fin proximal radial bone 3)] | count |  |
| pectoral fin proximal radial bone 3 | increased size |  |
| [articular surface and (part_of some pectoral fin proximal radial bone 2)] | count |  |
| pectoral fin proximal radial bone 2 | increased size |  |
| intermedium (fore) | cylindrical |  |
| ulnare | cuboid |  |
| pectoral fin | antero-posteriorly flattened |  |
| ulnar facet of the humerus | ovate |  |
| ulnar facet of the humerus | bulbous |  |
| [proximal region and (part_of some (autopod joint and (part_of some pectoral appendage)))] | convex |  |
| [distal region and (part_of some (autopod joint and (part_of some pectoral appendage)))] | concave |  |
| [anterior margin and (part_of some radius bone)] | sharp |  |
| [posterior margin and (part_of some radius bone)] | subcylindrical |  |
| ulna | cuboid |  |
| radial facet | ventral orientation |  |
| [pectoral fin lepidotrichium and (part_of some (distal region and (part_of some pectoral fin)))] | decreased size |  |
| [synovial joint and (part_of some ulnare)] | present |  |
| pectoral fin | increased thickness |  |
| ulnar facet of the humerus | ventral orientation |  |
| scapula | increased size |  |
| coracoid bone | increased size |  |
| radial facet | convex |  |
| glenoid fossa | concave |  |
| glenoid fossa | ovate |  |
| ulna | mobility |  |
| radial facet | elliptic |  |
| radius bone | elongated |  |
| [distal region and (part_of some radius bone)] | tapered |  |
| radius bone | arched |  |
| humeral facet on the ulna. | concave |  |
| humeral facet on the ulna. | decreased depth |  |
| [proximal region and (part_of some ulna)] | subrectangular |  |
| [ulnare and (continuous with some pectoral fin proximal radial bone)] | multiple |  |
| [skeletal joint and (distal_to some pectoral fin proximal radial bone)] | transverse orientation |  |
| [skeletal joint and (distal_to some pectoral fin distal radial bone)] | transverse orientation |  |
| [skeletal joint and (distal_to some proximal carpal bone)] | transverse orientation |  |
| [ventral surface and (part_of some ulnare)] | rugose |  |
| [ventral surface and (part_of some ulna)] | rugose |  |
| [ventral surface and (part_of some pectoral fin distal radial bone)] | rugose |  |
| radius bone | mobility |  |
| humeral facet on radius | concave |  |
| humeral facet on radius | ovate |  |
| [anterior margin and (part_of some proximal epiphysis of humerus)] | decreased width |  |
| [anterior margin and (part_of some proximal epiphysis of humerus)] | strap-shaped |  |
| cleithrum | ornamented |  |
| supracleithrum | ornamented |  |
| anocleithrum | ornamented |  |
| [facet and (part_of some proximal epiphysis of humerus)] | in contact with | glenoid fossa |
| [anterior region and (part_of some radial facet)] | aligned with | [ventral surface and (part_of some humerus)] |
| glenoid fossa | adjacent to | [ventral surface and (part_of some coracoid bone)] |
| [sulcus and (part_of some (ventro-medial region and (part_of some internal rim of coracoid foramen)))] | aligned with | [sulcus and (part_of some (dorso-medial region and (part_of some external rim of the coracoid foramen)))] |
| glenohumeral joint | decreased mobility |  |
| [anatomical projection and (part_of some (ventral surface and (part_of some pectoral fin distal radial bone)))] | present |  |
| [anatomical projection and (part_of some (ventral surface and (part_of some ulnare)))] | present |  |
| [anatomical projection and (part_of some (ventral surface and (part_of some ulna)))] | present |  |
| clavicle | decreased size |  |
| cleithrum | decreased size |  |
| supracleithrum | decreased size |  |
| anocleithrum | decreased size |  |
| scapula | increased height |  |
