# Supplementary material for: Data Sources for Trait Databases: Comparing the Phenomic Content of Monographs and Evolutionary Matrices
Source: PLoS One. 2016 May 18;11(5):e0155680. doi: 10.1371/journal.pone.0155680 (PMC4871461; doi:10.1371/journal.pone.0155680)
Supplement: S2 Appendix — (DOCX) [file pone.0155680.s002.docx]

Appendix S2. Percent character types in individual monographs and matrices.

| Publication | Type | Morphology | Neomorphic | Position | Number |
| --- | --- | --- | --- | --- | --- |
| Boisvert 2005 | monograph | 60.0 | 16.4 | 23.6 | 0.0 |
| Boisvert et al. 2008 | monograph | 52.9 | 0.0 | 41.2 | 5.9 |
| Boisvert 2009 | monograph | 30.2 | 16.0 | 51.9 | 0.9 |
| Coates 1996 | monograph | 57.4 | 12.7 | 22.5 | 7.4 |
| Garvey et al. 2005 | monograph | 56.9 | 14.7 | 26.7 | 1.7 |
| Shubin et al. 2006 | monograph | 52.0 | 10.4 | 33.6 | 4.0 |
| Shubin et al. 2014 | monograph | 70.4 | 16.7 | 11.1 | 0.0 |
| Carroll 2007 | matrix | 17.8 | 41.2 | 13.0 | 28.0 |
| Clack et al. 2012 | matrix | 40.8 | 46.5 | 9.9 | 2.8 |
| Daeschler et al. 2006 | matrix | 48.3 | 24.1 | 26.4 | 1.1 |
| Ruta 2011 | matrix | 48.4 | 34.9 | 13.5 | 3.2 |
| Swartz 2012 | matrix | 40.4 | 37.0 | 22.6 | 0.0 |
| Vallin and Laurin 2004 | matrix | 25.0 | 39.6 | 10.4 | 25.0 |
