# Supplementary material for: Data Sources for Trait Databases: Comparing the Phenomic Content of Monographs and Evolutionary Matrices
Source: PLoS One. 2016 May 18;11(5):e0155680. doi: 10.1371/journal.pone.0155680 (PMC4871461; doi:10.1371/journal.pone.0155680)
Supplement: S3 Appendix — (DOCX) [file pone.0155680.s003.docx]

Appendix S3. Breakdown of EQ complexity scores across EQ statements from matrices (A) and ‘monographs’ (B).

1. Matrix distribution of EQ complexity

| Carroll 2007 | Complexity score | Number of EQ's | Frequency (%) |
| --- | --- | --- | --- |
|  | 2 | 222 | 52.6 |
|  | 3 | 35 | 8.3 |
|  | 4 | 131 | 31.0 |
|  | 5 | 7 | 1.7 |
|  | 6 | 18 | 4.3 |
|  | 7 | 4 | 0.9 |
|  | 8 | 2 | 0.5 |
|  | 9 | 1 | 0.2 |
|  | 10 | 0 | 0.0 |
|  | 11 | 2 | 0.5 |
| Clack et al. 2012 | Complexity score | Number of EQ's | Frequency (%) |
|  | 2 | 50 | 72.5 |
|  | 3 | 2 | 2.9 |
|  | 4 | 14 | 20.3 |
|  | 5 | 1 | 1.4 |
|  | 6 | 2 | 2.9 |
|  | 7 | 0 | 0.0 |
|  | 8 | 0 | 0.0 |
| Daeschler et al. 2006 | Complexity score | Number of EQ's | Frequency (%) |
|  | 2 | 51 | 60 |
|  | 3 | 11 | 12.9 |
|  | 4 | 12 | 14.1 |
|  | 5 | 2 | 2.4 |
|  | 6 | 8 | 9.4 |
|  | 7 | 1 | 1.2 |
|  | 8 | 0 | 0.0 |
|  | 9 | 0 | 0.0 |
|  | 10 | 0 | 0.0 |
| Ruta et al. 2011 | Complexity score | Number of EQ's | Frequency (%) |
|  | 2 | 266 | 53.2 |
|  | 3 | 64 | 12.8 |
|  | 4 | 75 | 15 |
|  | 5 | 21 | 4.2 |
|  | 6 | 45 | 9 |
|  | 7 | 10 | 2 |
|  | 8 | 14 | 2.8 |
|  | 9 | 3 | 0.6 |
|  | 10 | 2 | 0.4 |
| Swartz et al. 2012 | Complexity score | Number of EQ's | Frequency (%) |
|  | 2 | 78 | 63.4 |
|  | 3 | 12 | 9.8 |
|  | 4 | 19 | 15.4 |
|  | 5 | 2 | 1.6 |
|  | 6 | 12 | 9.8 |
|  | 7 | 0 | 0.0 |
|  | 8 | 0 | 0.0 |
| Vallin and Laurin 2004 | Complexity score | Number of EQ's | Frequency (%) |
|  | 2 | 56 | 58.3 |
|  | 3 | 9 | 9.4 |
|  | 4 | 19 | 19.8 |
|  | 5 | 6 | 6.3 |
|  | 6 | 6 | 6.3 |
|  | 7 | 0 | 0.0 |
|  | 8 | 0 | 0.0 |

1. Monograph distribution of EQ complexity.

| Coates 1996 | Complexity score | Number of EQ's | Frequency (%) |
| --- | --- | --- | --- |
|  | 2 | 156 | 46.2 |
|  | 3 | 38 | 11.2 |
|  | 4 | 75 | 22.2 |
|  | 5 | 21 | 6.2 |
|  | 6 | 32 | 9.5 |
|  | 7 | 8 | 2.4 |
|  | 8 | 3 | 0.9 |
|  | 9 | 3 | 0.9 |
|  | 10 | 0 | 0.0 |
|  | 11 | 2 | 0.6 |
|  | 12 | 0 | 0.0 |
| Boisvert 2005 | Complexity score | Number of EQ's | Frequency (%) |
|  | 2 | 26 | 47.3 |
|  | 3 | 15 | 27.3 |
|  | 4 | 5 | 9.1 |
|  | 5 | 4 | 7.3 |
|  | 6 | 3 | 5.5 |
|  | 7 | 2 | 3.6 |
|  | 8 | 0 | 0.0 |
| Boisvert et al. 2008 | Complexity score | Number of EQ's | Frequency (%) |
|  | 2 | 18 | 35.3 |
|  | 3 | 11 | 21.6 |
|  | 4 | 10 | 19.6 |
|  | 5 | 8 | 15.7 |
|  | 6 | 0 | 0.0 |
|  | 7 | 1 | 2.0 |
|  | 8 | 2 | 3.9 |
|  | 9 | 1 | 2.0 |
|  | 10 | 0 | 0.0 |
| Boisvert 2009 | Complexity score | Number of EQ's | Frequency (%) |
|  | 2 | 47 | 44.3 |
|  | 3 | 24 | 22.6 |
|  | 4 | 12 | 11.3 |
|  | 5 | 12 | 11.3 |
|  | 6 | 6 | 5.7 |
|  | 7 | 5 | 4.7 |
|  | 8 | 0 | 0.0 |
| Garvey et al. 2005 | Complexity score | Number of EQ's | Frequency (%) |
|  | 2 | 40 | 34.5 |
|  | 3 | 25 | 21.6 |
|  | 4 | 25 | 21.6 |
|  | 5 | 7 | 6.0 |
|  | 6 | 9 | 7.8 |
|  | 7 | 6 | 5.2 |
|  | 8 | 2 | 1.7 |
|  | 9 | 0 | 0.0 |
|  | 10 | 0 | 0.0 |
|  | 11 | 0 | 0.0 |
|  | 12 | 0 | 0.0 |
|  | 13 | 0 | 0.0 |
|  | 14 | 0 | 0.0 |
|  | 15 | 2 | 1.7 |
| Shubin et al. 2006 | Complexity score | Number of EQ's | Frequency (%) |
|  | 2 | 58 | 47.2 |
|  | 3 | 13 | 10.6 |
|  | 4 | 29 | 23.6 |
|  | 5 | 6 | 4.9 |
|  | 6 | 10 | 8.1 |
|  | 7 | 3 | 2.4 |
|  | 8 | 1 | 0.8 |
|  | 9 | 0 | 0.0 |
|  | 10 | 0 | 0.0 |
|  | 11 | 3 | 2.4 |
|  | 12 | 0 | 0.0 |
| Shubin et al. 2014 | Complexity score | Number of EQ's | Frequency (%) |
|  | 2 | 24 | 45.3 |
|  | 3 | 6 | 11.3 |
|  | 4 | 16 | 30.2 |
|  | 5 | 3 | 5.7 |
|  | 6 | 2 | 3.8 |
|  | 7 | 2 | 3.8 |
|  | 8 | 0 | 0.0 |
